# Unbiased immunome characterisation correlates with COVID-19 mRNA vaccine failure in immunocompromised adults

**DOI:** 10.3389/fimmu.2024.1405217

**Published:** 2024-11-14

**Authors:** Juan H-Vázquez, Paloma Cal-Sabater, Elisa Arribas-Rodríguez, Aida Fiz-López, Candido Perez-Segurado, Álvaro Martín-Muñoz, Ángel De Prado, Marina Perez Mazzali, Carolina G. de Castro, Alejandro G. del Hierro, Ignacio de la Fuente Graciani, Sonia Pérez González, Sara Gutiérrez, Pablo Tellería, Cristina Novoa, Silvia Rojo Rello, Antonio Garcia-Blesa, Rosa Sedano, Ana María Martínez García, Sonsoles Garcinuño Pérez, Marta Domínguez-Gil, Cristina Hernán García, Ma Mercedes Guerra, Eduardo Muñoz-Sánchez, Cristina Barragan-Pérez, Soraya Diez Morales, Oriana Casazza Donnarumma, Daniel Ramos Pollo, Natalia Santamarta Solla, Paula Ma Álvarez Manzanares, Sara Bravo, Cristina García Alonso, Luis Alberto Avendaño Fernández, Jenifer Gay Alonso, José A. Garrote, Eduardo Arranz, José María Eiros, Fernando Rescalvo Santiago, Carolina Quevedo Villegas, Eduardo Tamayo, Antonio Orduña, Carlos Dueñas, María Jesús Peñarrubia, Sara Cuesta-Sancho, María Montoya, David Bernardo

**Affiliations:** ^1^ Mucosal Immunology Lab, Unit of Excellence Institute of Biomedicine and Molecular Genetics of Valladolid (IBGM, University of Valladolid-CSIC), Valladolid, Spain; ^2^ Flow Cytometry Facility, Unit of Excellence Institute of Biomedicine and Molecular Genetics of Valladolid (IBGM, University of Valladolid-CSIC), Valladolid, Spain; ^3^ Department of Hematology, Hospital Clínico Universitario de Valladolid, Valladolid, Spain; ^4^ Internal Medicine Department, Hospital Clínico Universitario de Valladolid, Valladolid, Spain; ^5^ Microbiology and Inmunology Unit, Hospital Clínico Universitario de Valladolid, Valladolid, Spain; ^6^ Microbiology Department, Hospital Universitario Río Hortega, Valladolid, Spain; ^7^ Servicio de Medicina Preventiva, Hospital Clínico Universitario de Valladolid, Valladolid, Spain; ^8^ Unidad de Cuidados Paliativos del Hospital Universitario Río Hortega, Valladolid, Spain; ^9^ Servicio de Prevención de Riesgos Laborales, Hospital Clínico Universitario de Valladolid, Valladolid, Spain; ^10^ Residencia y Centro de Día ORPEA Valladolid, Valladolid, Spain; ^11^ Unidad Investigación, Hospital Clínico Universitario de Valladolid, Valladolid, Spain; ^12^ Centro de Investigaciones Biomédicas en Red de Enfermedades Infecciosas (CIBERINFEC), Madrid, Spain; ^13^ Viral Immunology, Therapies and Vaccines Lab, Centro de Investigaciones Biológicas Margarita Salas (CIB), Madrid, Spain

**Keywords:** computational cytometry, vaccine failure, COVID-19, immunome, immunocompromised adult

## Abstract

**Introduction:**

Coronavirus disease 2019 (COVID-19) affects the population unequally, with a greater impact on older and immunosuppressed people.

**Methods:**

Hence, we performed a prospective experimental cohort study to characterise the effect of severe acute respiratory syndrome coronavirus 2 (SARS-CoV-2) vaccination in immune-compromised patients (older adults and oncohaematologic patients), compared with healthy counterparts, based on deep characterisation of the circulating immune cell subsets.

**Results and discussion:**

While acquired humoral and cellular memory did not predict subsequent infection 18 months after full vaccination, spectral and computational cytometry revealed several subsets within the CD8^+^ T-cells, B-cells, natural killer (NK) cells, monocytes and TEMRA Tγδ cells that were differentially expressed in individuals who were subsequently infected and not infected not just following immunisation, but also prior to vaccination. Of note, we found up to seven clusters within the TEMRA Tγδ cell population, with some of them being expanded and others decreased in subsequently infected individuals. Moreover, some of these cellular clusters were also related to COVID-19-induced hospitalisation in oncohaematologic patients. Therefore, we have identified a cellular signature that even before vaccination is related to COVID-19 vulnerability as opposed to the acquisition of cellular and/or humoral memory following vaccination with SARS-CoV-2 messenger RNA (mRNA) vaccines.

## Introduction

Coronavirus disease 2019 (COVID-19) has been shown to affect the population very unequally, with one of the main risk factors being a depressed immune system. In this regard, multiple types of COVID-19 vaccines have been shown to be highly effective not just in preventing severe acute respiratory syndrome coronavirus 2 (SARS-CoV-2) infection, but also in reducing post-infection symptoms. Indeed, all of these vaccines induce systemic immune responses, but little is known about their induced alterations in different immune cell subsets. Although the specific mechanisms of acquired humoral and cellular memory have been largely described, it has not been possible to relate those events to vaccine failure (1–4).

In this framework, defining the efficacy of SARS-CoV-2 vaccines in frail populations is of paramount relevance for the design and implementation of future vaccination strategies. However, little is known regarding long-term immunity responses triggered by SARS-CoV-2 vaccines in older people and patients with cancer after repeated booster doses (5, 6). Researchers have reported that patients with lymphoid cancers are particularly at risk of an inadequate antibody response to anti-SARS-CoV-2 vaccines (7), particularly those with non-Hodgkin lymphoma (NHL) receiving B-cell-depleting agents (8). In a similar manner, immunosenescence is probably one of the most relevant determinants of progression to severe COVID-19 (9), as ageing changes both adaptive and innate immunity, resulting in increased susceptibility to infections and development of chronic inflammation (10). Overall, vaccination is one of the most effective tool against COVID-19. Despite the success of COVID-19 vaccines – with their high efficacy in healthy populations – concerns about the efficacy and safety of these vaccines in immunocompromised populations remain unresolved (11, 12). Additionally, it is plausible that the pre-vaccination immune repertoire in each individual could play a crucial role in shaping the subsequent immune response towards vaccination, as it has been shown in the case of SARS-COV-2 viral infection (13).

As a consequence, there is an urgent need to better understand vaccine-induced immunogenicity in the context of heterogeneous host characteristics to improve protection for these patients by designing more efficient personalised vaccination regimes. To that end, we have performed a deep and unbiased characterisation of the circulating immune system (or immunome) using state-of-the-art spectral cytometry in immune-compromised patients (including older adults and oncohaematologic patients), compared with healthy counterparts. Our results have revealed that while the acquired humoral and cellular memory cannot prognosticate subsequent infection, unbiased analysis of the circulating immunome could predict subsequent infection even before vaccination. These findings pave the way for improving vaccination regimens for those patients.

## Results

### Cellular immunome identification

**Figure 1:**
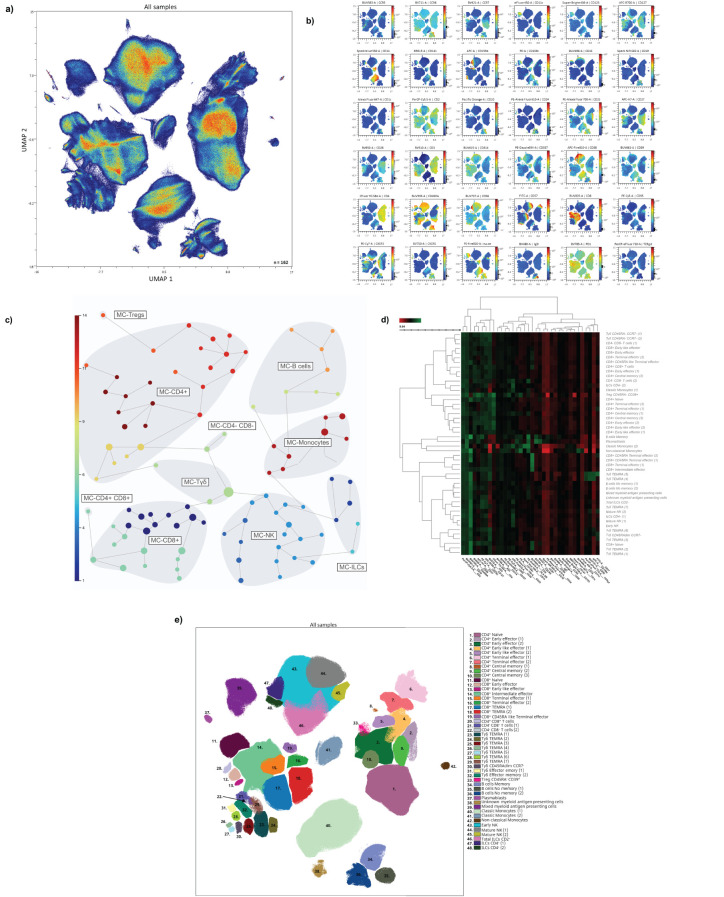
Immunome characterisation following UMAP analysis. **(A)** UMAP analysis was performed within total singlet viable CD45^+^ cells from all samples (n = 162). Subsequent down-sampling to a total of 4 × 10^6^ events was performed so that each cohort was equally represented. Surface expression intensities of the remaining 36 analysed markers are shown in **(B)**. The colour code is based on the intensity, where red represents higher expression and blue represents lower expression. **(C)** FlowSOM analysis of total singlet viable CD45^+^ cells identified the main metaclusters of the dataset: B-cells, NK cells, ILCs, Tγδ cells, T_regs_, CD4^+^ T-cells, CD8^+^ T-cells, CD4^+^CD8^+^ T-cells and CD4^–^CD8^–^ T-cells. **(D)** Heatmap displaying the intensity levels of each marker within the 48 identified clusters. The colour code is based on the expression intensity, where green represents higher expression and the transition to red represents lower expression. A dendrogram was generated by unsupervised hierarchical clustering. **(E)** All 48 identified clusters were overlaid on the UMAP projection (n = 162). Each identified cluster is tagged with a specific colour and number as shown in the legend.

Uniform manifold approximation and projection (UMAP) analysis of the 27 healthy adults, 20 older adults and 39 oncohaematologic patients at 2 different time points – before and after vaccination – (with a loss of 10 samples due to insufficient quality) identified four major continents and three smaller islands (**Figure 1A**). The relative expression of each marker from the UMAP analysis is shown in **Figure 1B**. The main continent on the left represents cytotoxic T-cells together with Tγδ cells, while the main continent on the right comprises helper T-cells. The smaller island on the bottom is mainly composed of B-cells and the two islands in the middle represent monocytes. Natural killer cells (NK) cells are in the top island together with innate lymphoid cells (ILCs), while the small top left island comprises mixed myeloid antigen-presenting cells.

To refine our analysis, we used the FlowSOM algorithm to find similar cell clusters and integrate them into metaclusters in an unsupervised manner (**Figure 1C**). We identified a total of 48 clusters according to the expression of the surface markers as shown in the heatmap (**Figure 1D**). **Table 1** shows an in-depth characterisation of the phenotype of all clusters, which allowed the identification of 46, because clusters 38 and 39 could not be clearly identified. In the case of cluster 38, although it is close to monocytes in the UMAP plot, there is no CD14 or CD16 expression. Hence, cluster 38 could be conventional dendritic cells (cDCs) as they are negative for almost every marker except HLA-DR – although they are also CD11c^–^, so their true nature remains elusive. Cluster 39 comprises intermediate (CD14^+^CD16^+^) and non-classical (CD14^+^CD16^–^) monocytes together with other HLA-DR^+^CD11c^+^ that could resemble cDCs.

**Table 1:** Cell cluster identification.

MC	Population	Subset	Phenotypic expression	Functional expression
1	CD4^+^	Naive	CD3, CD4, CD27, CD28, CD127, CD45RA	CCR7
2	CD4^+^	Early effector (1)	CD3, CD4, CD27, CD28, CD2dim, **CD25dim**	
3	CD4^+^	Early effector (2)	CD3, CD4, CD27, CD28, CD2dim, **CD127**	**CXCR3, FASdim**
4	CD4^+^	Early like effector (1)	CD3, CD4, CD28, CD2	**HLADRdim**
5	CD4^+^	Early like effector (2)	CD3, CD4, CD28, CD2, **CD127**	**Fasdim**
6	CD4^+^	Terminal effector (1)	CD3, CD4, CD2	**HNK1dim, FASdim**
7	CD4^+^	Terminal effector (2)	CD3, CD4, CD2	**CCR5dim**
8	CD4^+^	Central memory (1)	CD3, CD4, CD27**dim**, CD28, CCR7dim, **CD2dim, CD39, CD25dim, CD14dim, CD38**	**NKG2C, HLADRdim**
9	CD4^+^	Central memory (2)	CD3, CD4, CD27, CD28, CCR7dim, **CD127**	
10	CD4^+^	Central memory (3)	CD3, CD4, CD27, CD28, CCR7dim, **CD127**	**CXCR3**
11	CD8^+^	Naive	CD3, CD8, CD27, CD127, CD45RA, CCR7	CXCR3, NKG2Ddim
12	CD8^+^	Early effector	CD3, CD8, CD27dim, CD28, CD2, CD127	FASdim
13	CD8^+^	Early like effector	CD3, CD8, CD28, CD2, CD127	
14	CD8^+^	Intermediate effector	CD3, CD8, CD27dim, CD2, CD127	NKG2Ddim, CXCR3, FASdim
15	CD8^+^	Terminal effector (1)	CD3, CD8,CD2dim	NKG2Ddim
16	CD8^+^	Terminal effector (2)	CD3, CD8, CD2	NKG2Ddim, **HNK1dim**
17	CD8^+^	TEMRA (1)	CD3, CD8, CD2**dim**, CD45RA	NKG2D
18	CD8^+^	TEMRA (2)	CD3, CD8, CD2, CD45RA	NKG2D**dim, HNK1dim**
19	CD8^+^	CD45RA llike Terminal effector	CD3, CD8, CD2dim, CD45RAdim	NKG2A, NKG2Ddim, HNK1dim
20	CD4^+^/CD8^+^ T cells		CD3, CD4, CD8, CD27dim, CD28, CD2, CD127	FASdim
21	CD4^-^/CD8^-^ T cells	(1)	CD3, CD27**dim**, CD28dim, **CD127**	CCR5dim, **CXCR3dim**
22	CD4^-^/CD8^-^ T cells	(2)	CD3, CD27, CD28dim, **CD38, CD2dim**	CCR5dim**, FAS, HLADRdim**
23	Tγδ	TEMRA (1)	CD3, TCRγδ, CD45RA	
24	Tγδ	TEMRA (2)	CD3, TCRγδ, CD45RA, CD2dim, **CD8**	**NKG2D, FAS, PD1dim**
25	Tγδ	TEMRA (3)	CD3, TCRγδ**dim**, CD45RA, CD2dim	**HNK1dim**
26	Tγδ	TEMRA (4)	CD3, TCRγδ^++^, CD45RA, CD2dim, **CD56**	**NKG2D, NKG2A**
27	Tγδ	TEMRA (5)	CD3, TCRγδ^++^, CD45RA, CD2dim, **CD56**	**NKG2D, NKG2A, CXCR3dim, HNK1**
28	Tγδ	TEMRA (6)	CD3, TCRγδ, CD45RA, CD2dim, **CD127, CD27dim**	**CXCR3, NKG2D, NKG2A**
29	Tγδ	TEMRA (7)	CD3, TCRγδdim, CD45RA, CD2dim, **CD127, CD27dim**	**CXCR3**
30	Tγδ	CD45RAdim/CCR7^-^	CD3, TCRγδ, CD45RAdim, CD2dim	NKG2A, NKG2D, HNK1dim
31	Tγδ	Effector memory (1)	CD3, TCRγδdim, CD27, CD127, **CD28, CD2**	CXCR3, NKG2Ddim, CCR5dim, **NKG2A**
32	Tγδ	Effector memory (2)	CD3, TCRγδdim, CD27, CD127, CD2dim	CXCR3, NKG2Ddim, CCR5dim
33	Treg	CD45RA^-^/CD39^+^	CD3, CD4, CD27, CD28, CD25, CD39, CD2	FAS, NKG2C, HLADRdim, CXCR3, NKp30, CCR5
34	B cells	Memory	CD19, CD20, CD27, CD39, CD24, CD25, CD2, CD45RA, CCR7	CXCR3dim, NKG2C, CCR6, CXCR5, NKp30, HLA-DR
35	B cells	No memory (1)	CD20dim, CD28dim, CD39dim, CD2dim, IgD, CD45RA, **HLA-DR**	NKG2C, CXCR5, HLA-DRdim
36	B cells	No memory (2)	**CD19dim**, CD20dim, CD28dim, CD39dim, CD2dim, IgD, CD45RA**dim, CD24, CCR7, CD25dim**	NKG2C, CXCR5, HLA-DRdim, **CXCR3**
37	B cells	Plasmablasts	CD19, CD27, CD39, CD24, CD25, CCR7	NKG2C, CXCR5, HLADR
38	Unknown myeloid antigen presenting cells		CD45RA, CD16, CD39, CD11c, CD123, CD141, TCRγδdim, CD127, CD20dim	NKG2C, FASdim, HLA-DRdim, CXCR3
39	Mixed myeloid antigen presenting cells		CD45RA, CD16, CD39, CD11c, CD123, CD141	NKG2C, FASdim, HLA-DRdim
40	Monocytes	Classic (1)	CD14, CD38, CD39**dim**	FAS, NKG2C, HLA-DR**dim, CXCR5**
41	Monocytes	Classic (2)	CD14, CD38, CD39, **CD123**, **CD141, CD25, CD2, CD127, CD4dim**	FAS, NKG2C, HLA-DR, **NKp30**
42	Monocytes	Non-classical	CD16, CD39dim, CD123, CD141, CD25, CD2, CD11c^++^, CD1c, CD20dim, CD45RA	FAS, NKG2C, HLA-DR, NKp30, NKG2D
43	NK	Early NK	CD56, CD45RA, CD38	
44	NK	Mature NK (1)	CD56, CD45RA, CD38, CD16, CD2dim	HNK1dim, NKG2Ddim, **NKG2Adim**
45	NK	Mature NK (2)	CD56, CD45RA, CD38, CD16, CD2dim	HNK1dim, NKG2Ddim, **NKG2C**
46	ILCs	Total ILCs CD2^-^	CD56, CD38, CD127, CD45RA	CXCR3
47	ILCs	CD4^-^ (1)	CD56, CD38, CD127, CD39, CD2, **CD45RA**	CXCR3, NKG2A, NKG2Ddim, NKp30dim
48	ILCs	CD4^-^ (2)	CD56, CD38, CD127, CD39, CD2	CXCR3, NKG2A, NKG2Ddim, NKp30dim

For each of the 48 identified FlowSOM clusters (C), the cell population to which it belongs is shown, alongside the specific subset, phenotype and expression of functional markers. Markers highlighted in bold denote differential expression within the same population.

Of note, the same cell population can be divided into further subsets as shown in **Table 1** based on the expression of several surface markers. For example, TEMRA Tγδ (CD45RA**^+^** CCR7**^–^**) cells can be divided into 7 different populations based on the expression of the surface markers CD56, CD127, CD27 and CD8 together with NKG2D, FAS and CCR6, among others. Finally, we overlaid all of clusters onto the UMAP plot (**Figure 1E**) to determine not just how the relate one to each other, but also to display their pseudoevolution.

To validate these findings, we used the hierarchical or classical gating strategy to identify different immune cell subsets, as shown in **Supplementary Figure S1A**, with T-cells shown in **Supplementary Figure S1B**. Given that some of the identified clusters can be found within the same subset (**Table 1**; **Supplementary Figure S1D**), **Supplementary Figure S1C** displays the required gating strategy to identify these subsets while **Supplementary Figure S1D** shows the identification of the 7 TEMRA Tγδ cell subsets.

### In-depth immune characterisation of the cohorts at baseline

**Figure 2:**
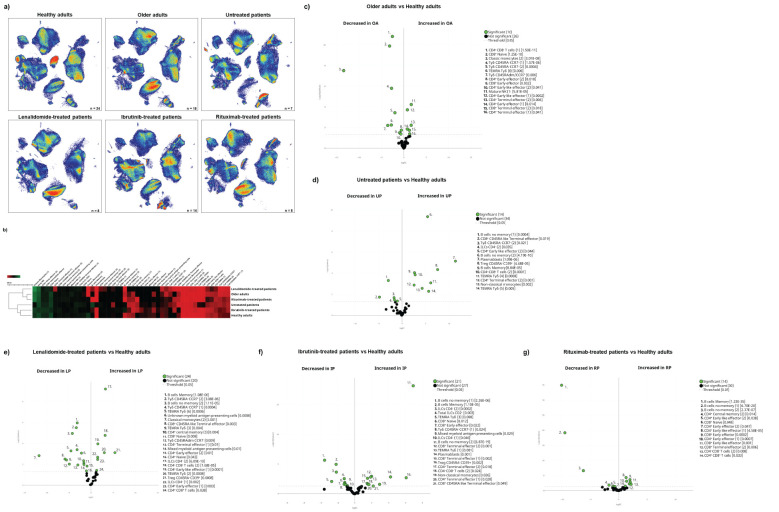
Cohort differences before vaccination. **(A)** The general UMAP plot displays the cohort distribution before vaccination, including healthy adults (n = 24), older adults (n = 18), untreated oncohaematologic patients (n = 7), lenalidomide-treated oncohaematologic patients (n = 8), ibrutinib-treated oncohaematologic patients (n = 14) and rituximab-treated oncohaematologic patients (n = 8). **(B)** The heatmap displays the intensity levels of each of the 48 identified clusters within the cohorts. The colour code is based on the expression intensity, where green represents higher expression and the transition to red represents lower expression. The dendrogram was generated by unsupervised hierarchical clustering. Volcano plots comparing the clusters identified in **Table 1** and **Figure 1** between healthy adults (n = 24) and **(C)** older adults (n = 18), **(D)** untreated patients (n = 7), **(E)** lenalidomide-treated oncohaematologic patients (n = 8), **(F)** ibrutinib-treated oncohaematologic patients (n = 14) and **(G)** rituximab-treated oncohaematologic patients (n = 8). The colour code is based on the expression intensity, where red represents higher expression and the transition to green represents lower expression. For the volcano plots, differentially expressed clusters (p < 0.05) in the comparisons are highlighted in green. Due to the low number of events, some clusters could not be analysed in **(C)** (CD45RA**^–^** CD39**^+^** T_regs_, non-classical monocytes, plasmablasts, CD4^–^CD8^–^ T-cells [2], TEMRA Tγδ cells [4] and TEMRA Tγδ cells [5]), **(D)** (non-classical monocytes, plasmablasts, TEMRA Tγδ cells [4] and TEMRA Tγδ cells [5]) and **(G)** (T_regs_ CD45RA**^–/^** CD39**^+^**, non-classical monocytes, plasmablasts and TEMRA Tγδ cells [4]).

Having described the global leucocyte subset composition (**Figure 1**), we next examined the differences between the cohorts at baseline (**Figure 2A**). The UMAP plot revealed a deficit of classical monocytes in older adults as well as in lenalidomide- and ibrutinib-treated oncohaematologic patients (clusters 40 and 41 in **Figure 1E**). As expected, rituximab-treated patients displayed a lack of B-cells (clusters 34–36 in **Figure 1E**), except for plasmablasts (cluster 37 in **Figure 1E**), of which there was a deficit in healthy adults, older adults and lenalidomide- and rituximab-treated oncohaematologic patients.

Differences according to surface marker expression within the cohorts are shown in the heatmap (**Figure 2B**). Older patients are closer to lenalidomide- and rituximab-treated oncohaematologic patients, while untreated and ibrutinib-treated oncohaematologic patients are closer to healthy adults. To validate and quantify these differences, we performed a paired comparison of each cohort relative to the healthy controls. We found that a third of the total identified clusters were differentially expressed among older adults and healthy controls: older adults displayed a significant expansion of CD4^+^ and CD8^+^ effector T-cells and a deficit of monocytes and immature T-cells (**Figure 2C**). To further confirm these results, we employed a classical gating strategy that revealed older adults have an expansion of mature NK cells together with several subsets of effector CD4^+^ and CD8^+^ T-cells, and a deficit of immature T-cells (mainly CD4^–^CD8^–^ T-cells and naïve CD8^+^ T-cells), CCR7^–^CD45RA^–^ Tγδ and plasmablasts (**Supplementary Figures S2A, B**). Moreover, and given that all clusters identified in **Figure 1** and **Table 1** do not always resemble a whole population as they can be further divided into subsets, we validated the specific clusters identified in **Figure 2C** by following the gating strategy displayed in **Supplementary Figures S1C, D**. Interestingly, even though early effector CD4^+^ T-cells did not differ between healthy and older adults, subset 1 of this population (cluster 2 in **Table 1**) was expanded in older adults, whereas its second subset (cluster 3 in **Table 1**) was reduced in older adults (**Supplementary Figure S2C**). Mature NK cell expansion in older adults (**Supplementary Figure S2A**) was specifically due to an expansion of subset 1 of mature NK (cluster 44 in **Table 1**), while the second subset of this population (cluster 45 in **Table 1**) remained unchanged. Therefore, this approach confirms the relevance of validating differences between as well as within different subsets.

We performed analyses for oncohaematologic patients based on their treatment status. Untreated oncohaematologic patients had 14 differentially expressed clusters compared with healthy adults (**Figure 2D**). Further analysis of this cohort revealed that these patients had a deficit of non-classical monocytes, TEMRA Tγδ cells, CD45RA^–^CD39^+^ regulatory T-cells (T_regs_) and early-like effector CD4^+^ T-cells, together with an expansion of terminal effector CD4^+^ T-cells and CD4^–^CD8^–^ T-cells (**Supplementary Figure S2D**). Moreover, when we performed this analysis based on the specific clusters (**Supplementary Figure S2E**), we found that subset 2 of early-like effector CD4^+^ T-cells (cluster 3 in **Table 1**) was reduced, while cluster 2 of the terminal effector CD4^+^ T-cells (cluster 7 in **Table 1**) was expanded. Further analysis also revealed a deficit of subset 1 of non-memory B-cells (cluster 35 in **Table 1**) in this population together with subset 2 of CD4^–^ ILCs (cluster 47 in **Table 1**). Finally, analysis of TEMRA Tγδ cells revealed that subset 2 of this population (cluster 24 in **Table 1**) was decreased while subset 5 (cluster 27 in **Table 1**) was expanded.

The lenalidomide-treated oncohaematologic patients displayed specific differences in half of the analysed clusters compared with the healthy controls (**Figure 2E**). Using the classical gating strategy, we found a loss in several CD4^+^ and CD8^+^ T-cell populations, together with non-classical memory B-cells, memory B-cells and CD45RA^–^CCR7^–^ Tγδ cells. On the contrary, these patients had an expansion of CD39^+^CD45RA^–^ T_regs_, TEMRA Tγδ cells, and CD4^–^ ILCs (**Supplementary Figures S2F, G**). Further analyses revealed the specific clusters responsible for these differences (**Supplementary Figure S2H**).

For ibrutinib-treated oncohaematologic patients, 44% of the clusters were differentially expressed compared with healthy adults (**Figure 2F**). Following hierarchical gating identification of populations, it was clear that these patients exhibited a deficit of memory B-cells, non-classical monocytes, naïve CD8^+^ T-cells, CD45RA^-^CD39^+^ T_regs_, CD45RA^–^CCR7^–^ Tγδ cells and total ILCs as well as CD4^–^ ILCs (**Supplementary Figure S2I**). On the other hand, they displayed an expansion of terminal effector CD4^+^ and CD8^+^ T-cells, early-like effector CD8^+^ T-cells, CD4^-^CD8^-^ T-cells and TEMRA Tγδ cells. The specific clusters responsible for these differences are shown in **Supplementary Figure S2J**.

Moreover, we examined differences between rituximab-treated oncohaematologic patients and healthy adults at baseline. The volcano plot shows a 29% difference in the total identified clusters including, as expected, total B-cell depletion (**Figure 2G**; **Supplementary Figure S2K**). In addition, these patients had a deficit of CD8^+^ naïve T-cells and an expansion of CD8^+^ early-like and terminal effector T-cells, together with CD45RA^–^CD39^+^ T_regs_. Further analysis revealed that although the CD4^+^ T-cell populations were not altered in this cohort, several of their specific clusters did show changes (**Supplementary Figure S2M**).

### Acquired humoral and cellular memory

**Figure 3:**
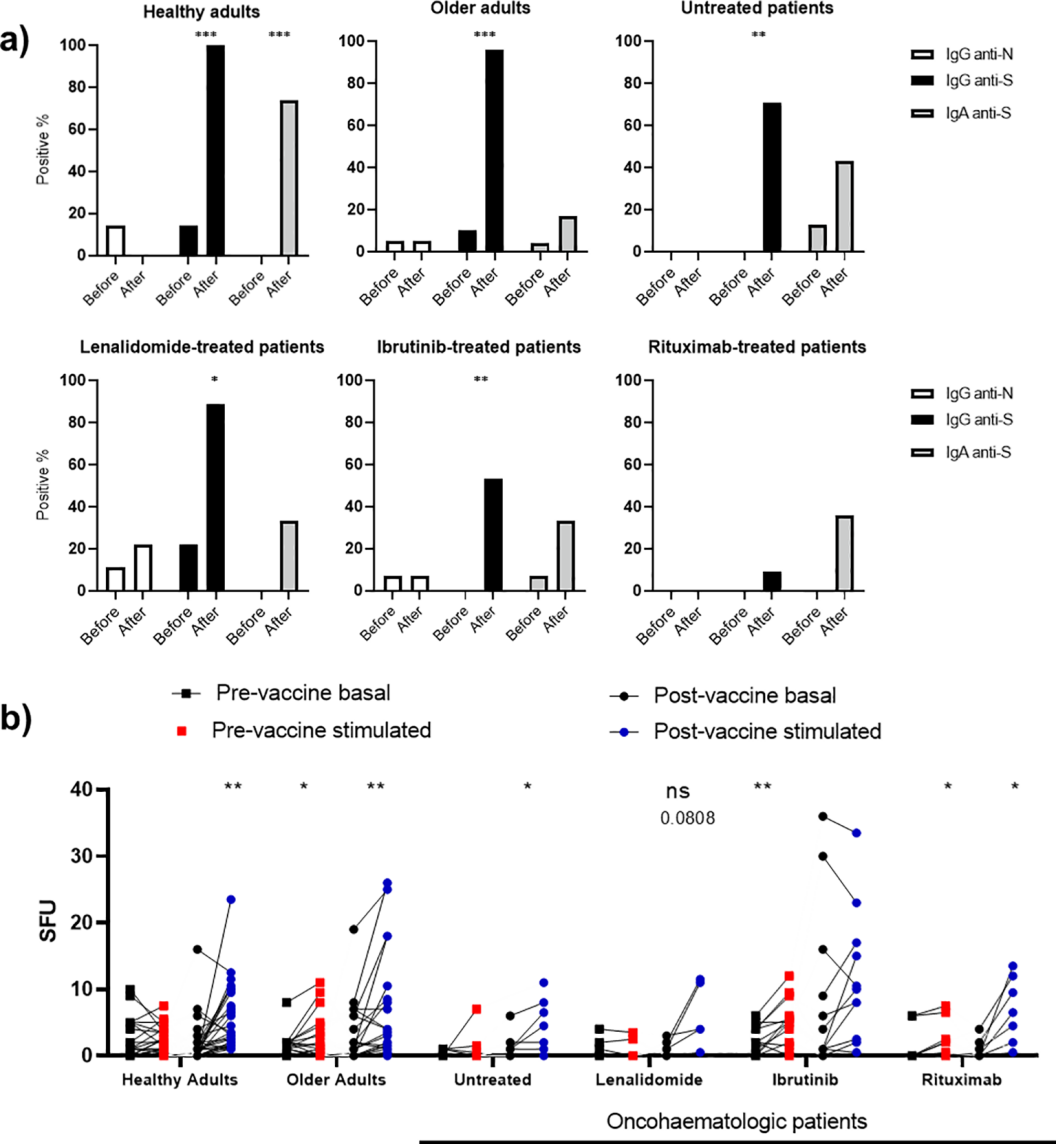
Vaccine-induced humoral and cellular memory. **(A)** Humoral memory against SARS-CoV-2 before and after vaccination. Anti-S IgG (black) and IgA (shaded) and anti-N IgG (white) were analysed. The results are based on the number of patients with positive serology. **(B)** Cellular memory against SARS-CoV-2 before and after vaccination analysed with an IFN-γ ELISpot assay. Each cohort was analysed independently by comparing the SFU under both basal (black dots) and SARS-CoV-2 peptide-stimulated (blue and red dots) conditions. Fisher’s exact test was applied in **(A)**, while a paired one-way ANOVA was applied in **(B)**. In all cases, p < 0.05 was considered significant (*p < 0.05; **p < 0.01; ***p < 0.001), while p < 0.10 was considered not significant (ns) but with a relevant trend (the exact p-value is shown).

Having described the cohorts at baseline, we next studied the acquired humoral and cellular immunity 3 months after full vaccination with messenger RNA (mRNA) vaccines. We assessed SARS-CoV-2 neutralising spike protein (S) antibodies (anti-S IgG and IgA) and nucleocapsid protein (N) antibodies (anti-N IgG) in plasma before and 3 months after full vaccination (two doses of the vaccine) (**Figure 3A**). A small fraction of healthy adults (14%), older adults (5%) and oncohaematologic patients treated with lenalidomide (11.1%) and ibrutinib (7%) had anti-N IgG antibodies before vaccination, suggesting an unnoticed previous asymptomatic infection. Of note, vaccination in healthy adults and older adults triggered anti-S IgG antibodies, confirming successful vaccination; however, the percentage was lower in oncohaematologic patients and virtually absent in rituximab-treated patients (**Figure 3A**). In addition, given that vaccination was intramuscular, IgA antibody production was mostly induced in healthy adults; it was not triggered in older adults or oncohaematologic patients. It is well known that vaccination efficiency in elderly individuals is often reduced due to immunosenescence. For example, the capacity of influenza vaccines to induce immune protection is age-related, with efficacy ranging from 70% to 90% in young people but decreasing to 30–50% in people over 65 years (14). Finally, we assessed induced cellular memory and found a strong response in all cohorts (including rituximab-treated patients), although it was smaller in lenalidomide-treated patients and absent in ibrutinib-treated patients (**Figure 3B**).

### Immunity-induced changes following vaccination

Having observed the differences before vaccination in all cohorts, we next analysed the differences before and after full vaccination (**Supplementary Figure S3A**). Healthy adults showed an expansion of the proportion of subset 3 of central memory CD4^+^ T-cells (cluster 10 in **Table 1**) and CD45RA^–^CD39^+^ T_regs_ (**Supplementary Figure S3B**). However, we could not validate this change with classical gating approaches.

In the case of older adults, vaccination induced changes in 15% of the clusters (**Supplementary Figure S3C**): it expanded the proportion of circulating CD4^–^CD8^–^ T-cells and early-like effector CD4^+^ T-cells and decreased classical and non-classical monocytes (**Supplementary Figure S3D**). Further analysis revealed that both subsets of classical monocytes decreased after vaccination while both CD4^–^CD8^–^ T-cell subsets, subset 2 of early effector CD4^+^ T-cells (cluster 3 in **Table 1**) and subset 2 of early-like effector CD4^+^ T-cells (cluster 5 in **Table 1**) were expanded (**Supplementary Figure S3E**).

For untreated oncohaematologic patients, vaccination decreased the levels of circulating memory B-cells and plasmablasts (**Supplementary Figure S3F**), although we could not confirm these changes with hierarchical gating approaches. On the contrary, vaccination induced changes in 4 clusters in the lenalidomide-treated patients (**Supplementary Figure S3G**), revealing a decrease in CD4^–^CD8^–^ T-cells and CD45RA^–^CD39^+^ T_regs_ after vaccination (**Supplementary Figure S3H**). The decrease in the CD4^–^CD8^–^ T-cells was due to a reduction in the second subset of this population (cluster 22 in **Table 1**) while these patients also expanded subset 5 of TEMRA Tγδ cells (cluster 27 in **Table 1**). For the ibrutinib-treated patients, vaccination decreased the levels of circulating monocytes (**Supplementary Figure S3J**), although we could not confirm this change with classical gating approaches. Finally, vaccination induced changes in 4 clusters of the rituximab-treated patients (**Supplementary Figure S3K**), confirming that these patients had a trend for a decreased level of subset 2 of the non-memory B-cells (cluster 36 in **Table 1**) following vaccination (**Supplementary Figure S3L**).

### Immune variations following vaccination correlate with SARS-CoV-2 infection

Having assessed the vaccine-induced immunity after vaccination, we performed a clinical follow-up of all individuals during an 18-month period. We found that 30.4% of the healthy adults displayed subsequent SARS-CoV-2 infection as defined by a positive PCR (**Table 2**). This percentage, however, was much lower in the older adults (15%) as they were protected in a nursing home environment. Finally, 41.12% of the oncohaematologic patients had an infection (1 untreated patient, 6 ibrutinib-treated patients, 1 lenalidomide-treated patient and 1 rituximab-treated patient). Of note, given the low number of infected patients, we considered them as a single cohort in the subsequent analyses (irrespective of their treatment).

**Table 2:** Patient demographics.

		Healthy adults (HA)	Older adults (OA)	Oncohematologic patients			
				Untreated (UP)	Lenalidomide-treated (LP)	Ibrutinib-treated (IP)	Rituximab-treated (RP)
n		27	20	7	8	14	10
**Age**		59 (50–63)	89 (86-94)	66 (62-70)	63.5 (56.25-74.75)	66 (59-71)	64 (60-73)
**Sex (female)**		15 (55.55%)	18 (90%)	5 (71.42%)	5 (62.5%)	5 (45.45%)	2 (20%)
**Vaccine**	BNT162b2 (Pfizer-BioNTech)	27	20	1	–	2	1
	mRNA-1273 (Moderna)	–	–	6	8	12	9
**Oncohematologic disease**	Chronic lymphocytic leukemia (CLL)	–	–	5	–	14	–
	Follicular Lymphoma (FL)	–	–	2	–	–	–
	non-Hodgkin’s Lymphoma	–	–	–	–	–	10
	Myeloma	–	–	–	8	–	–
**Treatment**	No treatment	–	–	7	–	–	–
	Lenalidomide	–	–	–	8	–	–
	Ibrutinib	–	–	–	–	14	–
	Rituximab	–	–	–	–	–	10
**SARS-CoV-2 PCR^+^**		7 out of 23	3 out of 20	1 out of 7	2 out of 7	9 out of 13	2 out of 7
**COVID-19 Disease (Severe)**		0 out of 7	0 out of 3	0 out of 1	0 out of 2	3 out of 9	0 out of 1

**Figure 4:**
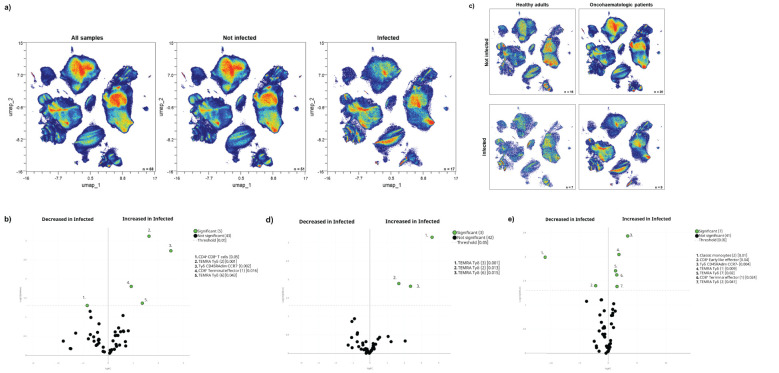
Cellular immunome post-vaccination predicts subsequent SARS-CoV-2 infection. **(A)** All samples following full vaccination (n = 68) are displayed in the UMAP density plots based on their subsequent infection (n = 17) defined by a positive PCR test. **(B)** Volcano plot comparing the clusters identified in **Table 1** and **Figure 1** based on subsequent infection (n = 17). **(C)** UMAP density plots of the infected and non-infected samples are displayed for healthy adults and oncohaematologic patients. Volcano plots comparing infected versus non-infected **(D)** healthy adults (n = 7 and n = 16, respectively) and **(E)** oncohaematologic patients (n = 9 and n = 20, respectively). For the UMAP plots, the colour code is based on the intensity, where red represents higher expression and blue represents lower expression. For the volcano plots, differentially expressed clusters (p < 0.05) in the comparisons are highlighted in green. Due to the low number of events, some clusters could not be analysed in **(D)** (plasmablasts, CD4**^–^** CD8**^–^** T-cells [2] and TEMRA Tγδ cells [4]).

The acquired humoral and cellular memory following immunisation did not predict subsequent infection for any of the analysed cohorts (**Table 3**). Nevertheless, when we evaluated the cellular immunome post-vaccination of all individuals in the context of subsequent infection, we found differences in the UMAP analysis (**Figure 4A**), specifically in 5 clusters (**Figure 4B**). Infected individuals had lower levels of CD4^+^CD8^+^ T-cells and a trend towards higher levels of TEMRA Tγδ cells and terminal effector CD8^+^ T-cells (**Supplementary Figure S4A**), due to an expansion in the second case of subset 1 (cluster 15 in **Table 1**) of this population (**Supplementary Figure S4B**). For a deeper insight into these observations, we performed additional analysis of healthy adults and oncohaematologic patients. However, given the low number of post-vaccinated infected older adults (n = 1), we excluded this cohort from the analysis.

**Table 3:** Infection based on humoral and cellular memory.

anti-N IgG		Number not infected	Number infected	p-value
Healthy adults	Negative serology	16	7	>0.9999
	Positive serology	0	0	
Older adults	Negative serology	14	3	>0.9999
	Positive serology	1	0	
Oncohaematologic patients	Negative serology	18	14	>0.9999
	Positive serology	1	0	
anti-S IgG		Number not infected	Number infected	p-value
Healthy adults	Negative serology	0	0	>0.9999
	Positive serology	16	7	
Older adults	Negative serology	0	0	>0.9999
	Positive serology	15	3	
Oncohaematologic patients	Negative serology	8	8	0.4905
	Positive serology	11	6	
anti-S IgA		Number not infected	Number infected	p-value
Healthy adults	Negative serology	5	1	0.6214
	Positive serology	11	6	
Older adults	Negative serology	12	3	>0.9999
	Positive serology	3	0	
Oncohaematologic patients	Negative serology	12	9	>0.9999
	Positive serology	7	5	
IFN-γ production		Mean	Standard error of the mean	p-value
Healthy adults	Not infected	5.125	0.936	0.4346
	Infected	7.071	3.087	
Older adults	Not infected	5.429	1.666	0.2258
	Infected	11.33	7.126	
Oncohaematologic patients	Not infected	7.211	1.175	0.2313
	Infected	11.13	4.142	

For each cohort, the absolute number of infected and non-infected individuals based on their serology status is shown for anti-N IgG and anti-S IgG and IgA. The mean and standard error for IFN-γ production following ELISpot assay towards S is also shown based on subsequent infection. The data were analysed with Fisher’s exact test (humoral memory) or a t-test (cellular memory). A p-value < 0.05 was considered statistically significant.

The UMAP analysis revealed visual differences between the infected and non-infected healthy adults and oncohaematologic patients (**Figure 4C**). Infected healthy adults had higher levels of 3 different TEMRA Tγδ cell subsets (**Figure 4D**), although we could not confirm these changes with hierarchical gating approaches. Infected oncohaematologic patients had differences in 15% of the total clusters (**Figure 4E**). Similarly to healthy adults, they showed an expansion of TEMRA Tγδ cells (**Supplementary Figure S4C**), specifically subsets 1, 2 and 7 (clusters 23, 24 and 29 in **Table 1**) of this population (**Supplementary Figure S4D**).

### The pre-vaccine immunome signature drives subsequent SARS-CoV-2 infection

Although vaccine-induce humoral and cellular immunity does predict subsequent infection, the circulating levels of the different TEMRA Tγδ cell subsets seem to anticipate it. Having said that, we cannot discard the possibility that these individuals were more exposed to the virus and thus were infected more often. Therefore, for a deeper insight into these mechanisms, we addressed whether these immune differences could be also observed before vaccination.

**Figure 5:**
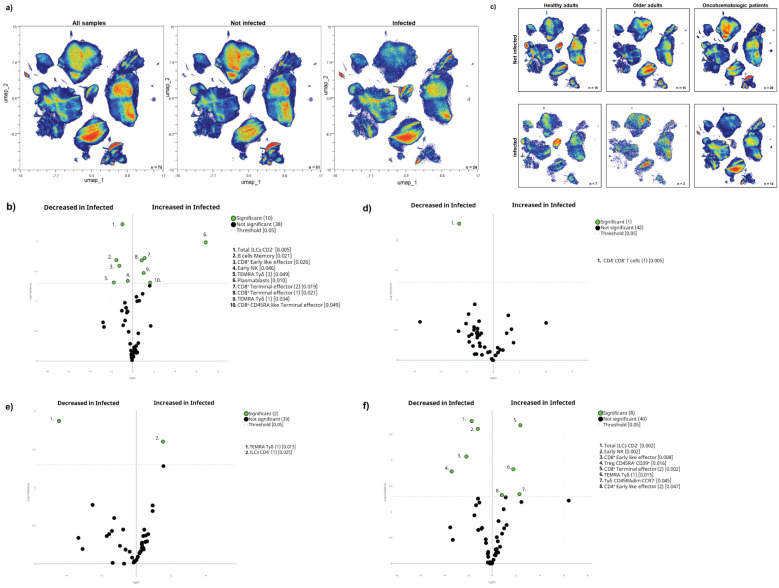
The pre-vaccination cellular immunome predicts SARS-CoV-2 infection. **(A)** All samples before the first vaccine dose (n = 75) are displayed in the UMAP density plots based on their subsequent infection. **(B)** A general volcano plot comparing the clusters identified in **Table 1** and **Figure 1** before vaccination based on subsequent infection. **(C)** UMAP density plots of the cohorts before being vaccinated are shown with the following colours: blue (healthy adults), purple (older adults) and green (oncohaematologic patients), relative to all samples (shown in black). Specific volcano plots of infected versus non-infected **(D)** healthy controls (n = 7 and n = 16, respectively), **(E)** older adults (n = 3 and n = 15, respectively) and **(F)** oncohaematologic patients (n = 14 and n = 20, respectively) are also shown. For the UMAP plots, the colour code is based on the intensity, where red represents higher expression and blue represents lower expression. For the volcano plots, differentially expressed clusters (p < 0.05) in the comparisons are highlighted in green. Due to the low number of events, some clusters could not be analysed in **(D)** (CD45RA**^–^**/CD39**^+^** T_regs_, non-classical monocytes, plasmablasts, CD4**^–^**/CD8**^–^** T-cells [2] and TEMRA Tγδ cells [4]) and **(E)** (classic monocytes [2], CD45RA**^–^**/CD39**^+^** T_regs_, non-classical monocytes, plasmablasts, CD4**^–^**/CD8**^–^** T-cells [2], TEMRA Tγδ cells [4] and TEMRA Tγδ cells [5]).

Indeed, we found differences in the immunome composition between subsequently infected and not-infected individuals, even before vaccination (**Figure 5A**) since 21% of the total clusters were differentially expressed between them (**Figure 5B**). Subsequently infected individuals had higher levels of circulating terminal effector CD8^+^ T-cells and plasmablasts, coupled with a trend towards higher levels of TEMRA Tγδ cells, and lower levels of early NK and total ILCs before vaccination (**Supplementary Figure S5A**). Further analysis confirmed that subset 3 of TEMRA Tγδ cells (cluster 25 in **Table 1**) was expanded in infected individuals even before vaccination (**Supplementary Figure S5B**).

After noting these differences, we performed additional analysis within the 3 cohorts (**Figure 5C**). Although healthy adults only had a difference in 1 cluster based on subsequent infection (**Figure 5D**), hierarchical gating revealed a trend towards higher levels of circulating plasmablasts and non-classical monocytes (**Supplementary Figure S5C**), with a deficit of subset 1 of CD4^–^CD8^–^ T-cells (cluster 21 in **Table 1**) and a trend towards lower levels of subset 4 of TEMRA Tγδ cells (cluster 26 in **Table 1**) in subsequently infected individuals (**Supplementary Figure S5D**). For older adults, only 2 clusters were differentially expressed before vaccination between subsequently infected and non-infected individuals (**Figure 5E**). For this cohort, there was a trend towards lower levels of subset 5 of TEMRA Tγδ cells (cluster 27 in **Table 1**) in subsequently infected individuals (**Supplementary Figure S5E**). Finally, it is clear that immunocompromised patients are more likely to get infected due to an overall reduced immune response. Indeed, 17% of the clusters were differentially expressed in the oncohaematologic patients (**Figure 5F**): these patients had higher levels of TEMRA Tγδ cells and a trend towards lower levels of early NK cells (**Supplementary Figure S5F**). Moreover, there was a specific expansion before vaccination of 1 of the TEMRA Tγδ cell subsets (cluster 23 in **Table 1**) in subsequently infected patients (**Supplementary Figure S5G**).

### Pre-vaccine immunome analysis in oncohaematologic patients could predict COVID-19-induced hospitalisation

**Figure 6:**
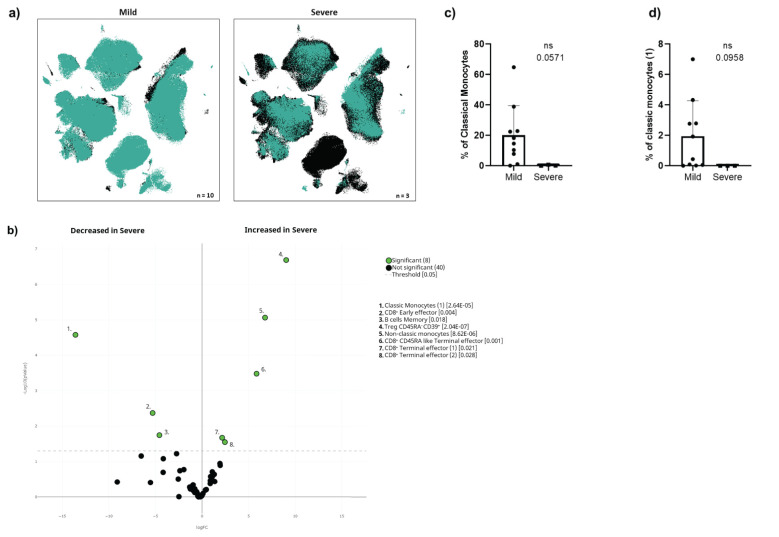
Cellular immunome before vaccination predicts COVID-19-induced hospitalisation. **(A)** UMAP plot of pre-vaccination samples of infected oncohaematologic patients according to the COVID-19 outcome defined as mild (left, no need for hospitalisation, n = 10) or severe (right, hospitalisation required, n = 3). **(B)** Volcano plot analysis comparing the clusters identified in **Table 1** and **Figure 1** for the infected oncohaematologic patients based on the COVID-19 outcome. **(C)** Classical validation, following the gating strategy shown in **Supplementary Figure S1**, of total monocytes, and **(D)** the classic monocyte (1) cluster. In **(B)**, green dots represent those clusters that showed significant differences (p < 0.05). A one-tailed t-test was applied for **(C)** and **(D)**; the p-values are shown in the figures (ns, not statistically significant).

We found that 3 of the 13 infected oncohaematologic patients had vaccine failure as defined by SARS-CoV-2-induced hospitalisation (**Table 2**). Thus, considering that the immunome composition before vaccination could predict subsequent infection, we addressed whether the same could be true within the oncohaematologic cohort to predict vaccine failure. The UMAP immunome analysis of this cohort divided into mild disease (no hospitalisation) and severe disease (required hospitalisation) displayed evident differences between them (**Figure 6A**). A total of 8 clusters were differentially expressed in these patients even before vaccination (**Figure 6B**). Patients with a severe outcome displayed an expansion of T_regs_, non-classical monocytes and CD8**^+^** effector T-cells. However, we could not confirm these findings with classical gating approaches, likely due to the low number of patients with a severe outcome (data not shown). Nevertheless, patients with a severe outcome displayed a trend towards a deficit of classical monocytes (**Figure 6C**) due to a deficit of subset 1 (cluster 40 in **Table 1**) (**Figure 6D**), confirming the relevance of this cell type to control subsequent infection.

## Discussion

For the past 3 years of the COVID-19 pandemic, the immune system of the vast majority of humans has come into contact with SARS-CoV-2 through vaccination, infection or both. Vaccination has been crucial to contain the impact of the COVID-19 pandemic (15). New evidence suggests that ‘hybrid’ immunity, as a result of both vaccination and natural infection, can provide partial protection against reinfection for at least 8 months (16). However, long-term immune protection has proved to be more complex than initially suggested. To date, only two systematic reviews have provided meta-analytical evidence on the duration of COVID-19 vaccine effectiveness (17, 18). Both reviews revealed a general decrease in vaccine effectiveness over time against infections, hospitalisations and mortality. This seems especially true for the humoral response elicited by mRNA vaccines, which can be escaped by variants of concern, rather than for T-cell-mediated responses (19). The adaptive immune response is known to play an important role in viral clearance, disease containment and resolution (20, 21). However, a study of immunocompromised patients, either due to disease or age, has not yet been performed. Therefore, in this work we have described for the first time, to our knowledge, the changes induced in the circulating immune system following vaccination of these patients and how that related to subsequent infection.

To get a deeper insight into the shape and status of acquired immunity in immunocompromised individuals, we performed an unbiased characterisation of the immune system before and after immunisation. Although the acquired levels of humoral and cellular memory could not predict subsequent infection, the immunome analysis showed differences not just in subsequent PCR-confirmed infection following immunisation, but also prior to vaccination. Nevertheless, it is true that PCR was not systemically done on a regular basis for all the individuals during the entire length of this study. Therefore, we do not know if the so-called non-infected patients were actually never infected by SARS-CoV-2 or if they were infected by the virus, but were more efficient at controlling the infection and thus did not develop any symptoms and did not receive a PCR test. Nevertheless, an obvious consequence of vaccine failure is COVID-19-induced hospitalisation that, in our case, was restricted to the ibrutinib-treated oncohaematologic patients, revealing a unique immune fingerprint in these patients even before vaccination. Of note, these patients had a complete depletion of both classical monocytes and subset 1 of these cells (cluster 40 in **Table 1**). Hence, monocyte depletion might disrupt the cross-talk between innate and adaptive immune cells, weakening the overall immune response. Monocytes also play a role in modulating B-cell responses, and their absence might affect the quality of antibody production post-vaccination, thereby compromising humoral memory. This interaction between the innate (monocyte) and adaptive (T- and B-cell) immune systems could be a critical factor in determining vaccine efficacy, particularly in patients with underlying immunosuppression. Therefore, future studies should study monocytes in general, and CXCR5^+^NKp30^–^ monocytes in particular (as they characterise this specific cluster), to unravel their specific contribution conferring vaccine protection even before that.

We are aware that this is a pilot study with a small sample size and that further characterisation is required with a larger sample size. It is important to highlight that while the computational cytometric analysis pipeline revealed several differentially expressed clusters in the volcano plots, we could not always validate these results with hierarchical gating approaches. This outcome is likely due to low number of available samples because many of the comparisons displayed a trend for a significant difference. Therefore, we cannot discard the possibility that most of the clusters identified in the volcano plots could have been validated by classical gating approaches if we had enrolled more individuals in this pilot study. In this context, it is important to note that in complex immunological studies, particularly those involving heterogeneous or rare cell populations, classical gating may not always capture the full spectrum of cellular responses. This limitation can result in an incomplete interpretation of immune response data. To achieve accurate interpretation, it is crucial to supplement classical gating with more advanced and flexible analysis techniques, such as computational approaches, which allow for a more comprehensive exploration of the immune response. This ensures that the conclusions drawn from the data accurately reflect the true complexity of immune responses across all patient cohorts. That said, the cell populations and subsets we validated using classical hierarchical approaches further strengthen our findings. In this regard, the use of unsupervised analyses revealed the presence of several clusters within a given population that would have otherwise remained undetected. A clear example of this phenomenon is the TEMRA Tγδ cell population, which has up to 7 subsets based on the differential expression of several surface markers (**Table 1**; **Supplementary Figure S1D**). It is important to highlight that several of its subsets could predict subsequent PCR-confirmed infection in the different cohorts, not just following vaccination but also before it. For example, subset 1 of this population (cluster 23 in **Table 1**) was expanded in subsequently infected oncohaematologic patients both before and following vaccination. Similarly, subsets 2 (cluster 24 in **Table 1**) and 7 (cluster 29 in **Table 1**) were also expanded in oncohaematologic patients before vaccination, while subsets 4 (cluster 26 in **Table 1**) and 5 (cluster 27 in **Table 1**) decreased in healthy and older adults, respectively, before vaccination. These findings confirm the relevance of the different TEMRA Tγδ cell populations to control SARS-CoV-2 infection. TEMRA γδ T cells may therefore provide early and robust responses to infection, potentially offering protection even before the full activation of classical adaptive immunity. This makes them especially relevant for immunocompromised individuals. The ability of γδ T cells to respond independently of MHC presentation may help compensate for the weakened T and B cell responses seen in patients treated with immunosuppressive drugs, such as ibrutinib and lenalidomide. Given the capacity of TEMRA γδ T cells to act quickly and independently of traditional antigen presentation mechanisms, we hypothesise that these cells may serve as critical first responders to vaccination in immunocompromised individuals. Therefore, these results suggest that this population and its subsets should be analysed further to understand the specific mechanisms by which they control SARS-CoV-2 infection. Indeed, they could also be considered as novel biomarkers to monitor and predict infection in immune-compromised individuals.

More generally, an important consideration when studying vaccine responses is whether the individuals had had prior immunologic encounters. For example, prior mild SARS-CoV-2 infection followed by complete clinical recovery sets up individuals, particularly men, to mount a more robust response to subsequent flu vaccination (22). This phenomenon is due to long-lasting antigen-agnostic trained innate immunity mechanisms (23) and bystander activation (not SARS-CoV-2 specific) of virtual memory (VM) and VM-like CD8+ T-cells (24). As such, any immune challenge may establish new baseline immune statuses with the potential to impact future responses in both antigen-specific and antigen-agnostic ways (25). In this regard, our findings provide additional insights into these mechanisms as we also observed the potential to predict vaccine failure even before vaccination, opening the way for specific treatment to those patients.

In addition to the inherent properties of vaccines, other factors can contribute to their overall effectiveness, such as sex, age, co-morbidities, pre-existing diseases or socio-economic background (26–29). Throughout the lifespan, sex and age are fundamental transformers of immunity to infectious diseases and to the response to vaccination (30). Nevertheless, we did not have enough data to segregate based on sex; this issue needs to be addressed in future studies.

In summary, we have shown that although vaccine-induced humoral and cellular memory cannot predict subsequent infection in immune-compromised patients, an unbiased characterisation of the circulating immunome correlates with vaccine outcome even before vaccination. Of course, additional research is needed to establish the robustness and reliability of these predictions. Future studies should expand this pilot study by focussing on the relevance of the already identified cell populations that seem to play a pivotal role controlling SARS-CoV-2 infection, with a particular focus on the TEMRA Tγδ cells. These studies will pave the way for personalised vaccination in the vulnerable population.

## Materials and methods

### Patient cohorts

To evaluate whether mRNA vaccines are equally effective in immune-compromised patients, two cohorts of these patients were recruited, including older adults and patients with lymphoid cancer (oncohaematologic patients); these cohorts were subsequently compared with a control cohort. In all cases, individuals with a previous PCR-confirmed diagnosis of COVID-19 were excluded from the study. Older adults (2 males, 18 females) were recruited from the Orpea residential nursing home (Valladolid, Spain). The mean age of the older adults was 88.1 years (all over 70 years old). All of them had been vaccinated with BNT162b2 (Pfizer-BioNTech). Thirty-nine oncohaematologic patients treated at the Department of Hematology (Hospital Clínico Universitario de Valladolid, Valladolid, Spain) were also recruited. Seven of them had chronic lymphocytic leukaemia (CLL) or follicular lymphoma without active treatment, 10 had NHL treated with rituximab, 14 had CLL treated with ibrutinib and 8 had myeloma treated with lenalidomide. With the exception of 4 patients vaccinated with BNT162b2 (Pfizer-BioNTech), the remaining were vaccinated with mRNA-1273 (Moderna). This cohort showed homogeneity in terms of sex and had an average age of 63.5 years. Finally, a total of 27 healthy controls with no known inflammatory, autoimmune or malignant diseases were recruited from the occupational risk prevention service (Hospital Clínico Universitario de Valladolid, Valladolid, Spain). The controls were age- and sex-matched to the other cohorts. All of them were vaccinated with BNT162b2 (Pfizer-BioNTech). An 18-month clinical follow-up was performed in all individuals to further address (following vaccination) subsequent PCR-confirmed SARS-Cov-2 infection. Additional information about patient demographics and subsequent infection can be found in **Table 2**. Ethics approval was obtained from the local ethics committee from Valladolid Este (PI 21-2098).

### Biological samples

Blood samples from all individuals were obtained before the first vaccine dose (between January and April 2021) and 3 months following full vaccination (i.e., 3 months after the second vaccine dose). Hence, the SARS-CoV-2 B.1.1.7 strain was predominant in Spain at the time of the pre-vaccine samples while the B.1.617.2 strain was predominant by the time that the post-vaccination sample was obtained.

In all cases, blood was collected in LH Lithium Heparin separator tubes. Subsequently, peripheral blood mononuclear cells (PBMCs) were isolated using Cytiva Ficoll-Paque™ PLUS (Cytiva 17-1440-03). Blood was slowly poured into a centrifuge tube with Ficoll-Paque™ without mixing (3 ml of Ficoll for 5 ml of blood) and centrifuged at 800 g for 30 min at 4°C (Fisherbrand™ GT2) with acceleration set to maximum and deceleration to minimum. PBMCs were collected from the interface between the Ficoll-Paque™ and plasma layers. PBMCs were centrifuged again in RPMI at 400 g for 5 min at 4°C to wash them. The resulting pellet was suspended in freezing medium (90% foetal bovine serum [FBS] + 10% dimethyl sulphoxide [DMSO]) to cryopreserve the cells in liquid nitrogen until use. Plasma samples were also obtained and immediately preserved at -80°C until use.

### Humoral memory

The determination of specific anti-S IgG and IgA antibodies was performed by electrochemiluminescence immunoassay (Elecsys Anti-SARS-CoV-2 S, Roche Diagnostics, Mannheim, Germany). The results are expressed in binding antibody units (BAU). In addition, the presence of anti-N IgG antibodies was evaluated with an enzyme-linked immunosorbent assay (ELISA) (COVID-19 ELISA IgG, Vircell Microbiologists, Santa Fe, Granada, Spain).

### Cellular memory

The magnitude and kinetics of the cellular response to SARS-CoV-2 was tested with an *ex vivo* IFN-γ ELISpot assay. PBMCs (both before vaccination and 3 months following full vaccination) were thawed in sterile tubes with 10 ml of RPMI 1640 without L-glutamine (Gibco, Paisley PA49RF, Scotland, United Kingdom) and centrifuged at 400 g for 5 min at 4°C. After removal of the supernatant, 1 ml of AIM-V serum-free medium with L-glutamine, 50 μg/ml streptomycin sulphate and 10 μg/ml gentamycin sulphate (Gibco) was added to count the cells in a BLAUBRAND^®^ Neubauer counting chamber in the presence of trypan blue. Cells were cultured in duplicate (100,000 viable cells in 200 μl of AIM-V medium) in 96 U-bottom culture plates for a total period 48 h in the presence of 2 μg/ml of a pool of SARS-CoV-2 spicule S1 domain peptides (Mabtech). As a positive control, total PBMCs were stimulated with a polyclonal stimulus of anti-CD3 and anti-CD28 (Mabtech), at concentrations of 0.2 and 0.02 μg/ml, respectively; unstimulated cells served as a negative control. Following culture, secreted interferon gamma (IFN-γ) was detected by adding 1 μg/ml anti-IFN-γ monoclonal antibody (7-B6-1-ALP, Mabtech) and incubating for 2 h in the dark. The plates were developed using BCIP/NBT-plus, according to the manufacturer’s instructions. The results were obtained as spot-forming units (SFU).

### Antibody staining and spectral cytometry acquisition

After thawing, and in parallel to determining cellular memory, 2 × 10^6^ PBMCs were stained with monoclonal antibodies (**Supplementary Table S1**) to be subsequently characterised by spectral cytometry (CyTek Aurora 5-laser) following the OMIP-069 protocol and analysis panel, with slight variations (31).

Briefly, before staining the PBMCs, the Live/Dead Fixable Blue Dead Cell Stain Kit (Molecular Probes, Thermo Fisher Scientific) was added to exclude dead cells from the analysis. Brilliant Stain Buffer and True-Stain Monocyte Blocker were also added prior to staining with the antibodies to obtain optimal fluorescence of the desired cells. The PBMCs were washed with fluorescence-activated cell sorting (FACS) buffer (500 ml phosphate-buffered saline [PBS] + 10 ml filtered FBS + 0.1 g NaN_3_ + 2.5 ml sterile ethylenediaminetetraacetic acid [EDTA]) and incubated in the dark at room temperature during the staining process. Finally, the cells were fixed with 0.8% paraformaldehyde in FACS buffer in the dark for 10 min, washed with FACS buffer and stored at 4°C. Cells were acquired within 48 h in a 5-laser spectral cytometer (Aurora, Cytek).

### Computational cytometric analysis and statistical analysis

The OMIQ Data Science platform (Omiq, Inc. 2022) was used following transformation of the data; the scale, parameters and cofactors were set as suggested by the platform. The data-cleaning FlowAI algorithm was applied to remove outlier events in spectral cytometry data files due to abnormal flow behaviour resulting from clogs and other common technical problems. Subsequently, a manual discard was performed to eliminate cell debris and doublets and to select viable leucocytes (CD45^+^ cells), which were used for subsequent analysis. After cleaning, a total of 10 samples did not fulfil the required quality criteria, so they were excluded from the analysis (3 pre-vaccine healthy adults, 2 pre-vaccine older adults, 3 post-vaccine ibrutinib-treated patients and 2 pre-vaccine rituximab-treated patients).

Due to the large amount of data obtained with this panel, it is not advisable to examine the results exclusively through traditional manual identification due to their subjectivity. Therefore, an unsupervised approach applying the UMAP algorithm was used for the exploratory analysis. Briefly, this algorithm uses a non-linear method based on graphs to represent information in multiple dimensions, and then reconstructs the results into a two-dimensional map, preserving the multidimensional structure. In this way, the algorithm finds similarities between cells in all dimensions. These dimensions are, in this case, the intensity of the markers that they express. The algorithm returns a two-dimensional map where the proximity of cells reflects their distances in multidimensional space, such that cells with similar patterns of expression are located very close to each other. This distance/similarity relationship is respected within and between each group or islet. A prior subsampling or random selection of events was performed until a total of 4 × 10^6^ events was reached to ensure that each cohort was equally represented.

Subsequently, the FlowSOM algorithm was used to find similar cell clusters and to separate them into groups in an unsupervised manner. This algorithm analyses the expression of all the selected markers in each of the cells of each sample and then groups them into metaclusters according to their expression level. In this way, it not only allows for the visualisation of cells in typical biological groupings, but also for the detection of new or unexpected clusters. However, this algorithm only displayed metaclusters that would represent the large subsets of the immune system present in the sample. The visual representation of the two algorithms allows one to further to subdivide these metaclusters into clusters that provide a more accurate representation of all the phenotypic and functional subsets of the human immunome. A clustered heatmap was created using the clusters obtained in the previous point. This heatmap graphically represents the level of expression of each phenotypic marker into each cluster. Dendrograms grouped clusters and phenotypic markers associated by similarity (distance). This approach permits one to identify the immune cell subsets represented by each cluster based on the expression levels of their markers. In this way, if a specific cluster is associated with a condition under study, its phenotype could be elucidated to identify it using classical supervised approaches where it would otherwise have gone undetected. Finally, the refined results of the FlowSOM algorithm were mapped on the UMAP plot to observe their distribution.

### Statistical analyses

For the computational cytometric data, volcano plots were constructed with the edgeR algorithm to compare cluster differences. Once the clusters showing significant differences were identified, the data were validated with classical hierarchical analysis. Using a modified gating strategy of the OMIP-69 panel, the percentages within the total viable leucocyte fraction (CD45^+^) of those clusters that stood out in the previous analysis were obtained, and then GraphPad Prism 9 (GraphPad Software, San Diego, CA, USA) was used for statistical analysis. Quantitative variables are expressed as mean and standard deviation (because they followed a normal distribution). One-Way analysis of variance (ANOVA), Fisher’s exact test and t-test comparisons were applied as detailed in each figure legend. In all cases, p < 0.05 was considered statistically significant.

## Data Availability

The original contributions presented in the study are included in the article/**Supplementary Material**. Further inquiries can be directed to the corresponding author.

## Supplementary material

Supplementary Table 1 Monoclonal antibodies used in this study.
