# Supplementary material for: Unbiased immunome characterisation correlates with COVID-19 mRNA vaccine failure in immunocompromised adults
Source: Front Immunol. 2024 Nov 14;15:1405217. doi: 10.3389/fimmu.2024.1405217 (PMC11602488; doi:10.3389/fimmu.2024.1405217)
Supplement: Supplementary Table 1 — Monoclonal antibodies used in this study. [file DataSheet1.docx]

**SUPPLEMENTARY MATERIAL**

| **Specificity** | **Fluorochrome** | **Clone** | **Supplier** |
| --- | --- | --- | --- |
| CCR5 | BUV563 | 2D7/CCR5 | BD |
| CCR6 | BV711 | G034E3 | BioLegend |
| CCR7 | BV421 | G043H7 | BioLegend |
| CD11c | eFluor 450 | 3.9 | ebiosience |
| CD123 | Super Bright 436 | 6H6 | ebiosience |
| CD127 | APC-R700 | HIL-7R-M21 | BD |
| CD14 | Spark Blue 550 | 63D3 | BioLegend |
| CD141 | BB515 | 1A4 | BD |
| CD159a (NKG2a) | APC | REA110 | Miltenyi |
| CD159c (NKG2c) | PE | REA205 | Miltenyi |
| CD16 | BUV496 | 3G8 | BD |
| CD19 | Spark NIR 685 | HIB19 | BioLegend |
| CD1c | Alexa Fluor 647 | L161 | BioLegend |
| CD2 | PerCP-Cy5.5 | TS1/8 | BioLegend |
| CD20 | Pacific Orange | HI47 | Thermo Fisher |
| CD24 | PE-Alexa Fluor 610 | SN3 | Thermo Fisher |
| CD25 | PE-Alexa Fluor700 | CD25-3G10 | Thermo Fisher |
| CD27 | APC-H7 | M-T271 | BD |
| CD28 | BV650 | CD28.2 | BioLegend |
| CD3 | BV510 | SK7 | BioLegend |
| CD314 (NKG2d) | BUV615 | 1D11 | BD |
| CD337 (NKp30) | PE-Dazzle594 | P30-15 | BioLegend |
| CD38 | APC-Fire810 | HIT2 | BioLegend |
| CD39 | BUV661 | TU66 | BD |
| CD4 | cFluor YG584 | SK3 | Cytek |
| CD45 | PerCP | HI30 | BioLegend |
| CD45RA | BUV395 | 5H9 | BD |
| CD56 | BUV737 | NCAM16.2 | BD |
| CD57 (HNK1) | FITC | HNK-1 | BioLegend |
| CD8 | BUV805 | SK1 | BD |
| CD95 (FAS) | PE-Cy5 | DX2 | BioLegend |
| CXCR3 | PE-Cy7 | G025H7 | BioLegend |
| CXCR5 | BV750 | RF8B2 | BD |
| HLA-DR | PE-Fire810 | L243 | BioLegend |
| IgD | BV480 | IA6-2 | BD |
| PD-1 | BV785 | EH12.2H7 | BioLegend |
| TCRγδ | PerCP-eFluor 710 | B1.1 | ebiosience |
| Viability | Live Dead UV Blue |  | Invitrogen |

**Supplementary Table 1. Monoclonal antibodies used in this study.**

**
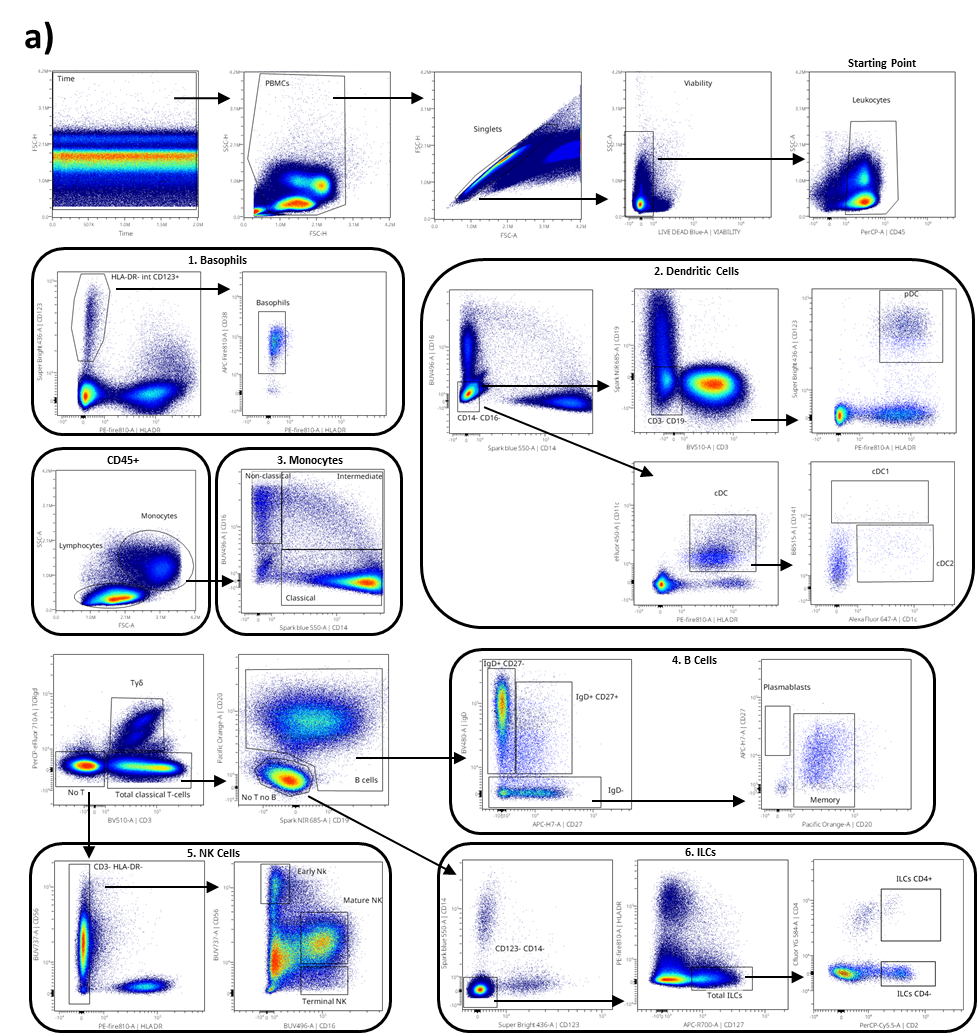

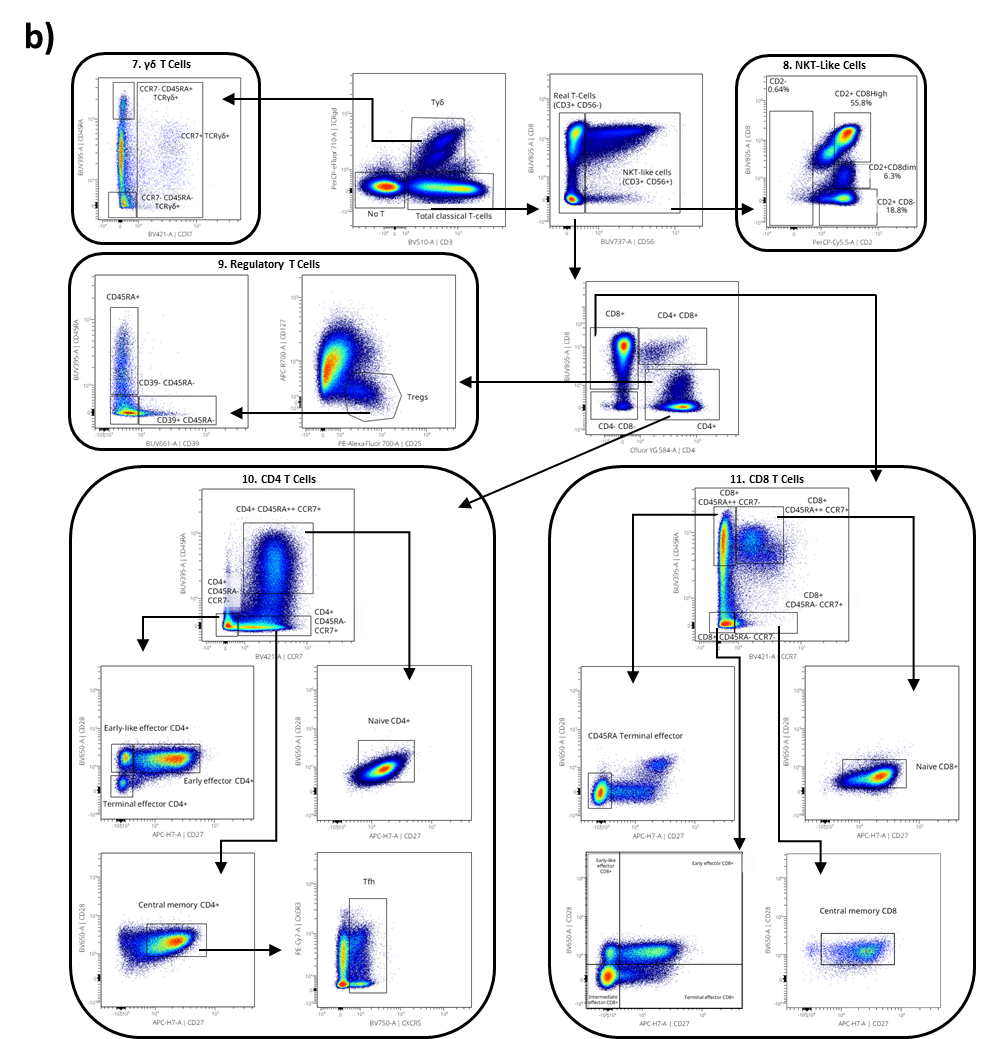
**


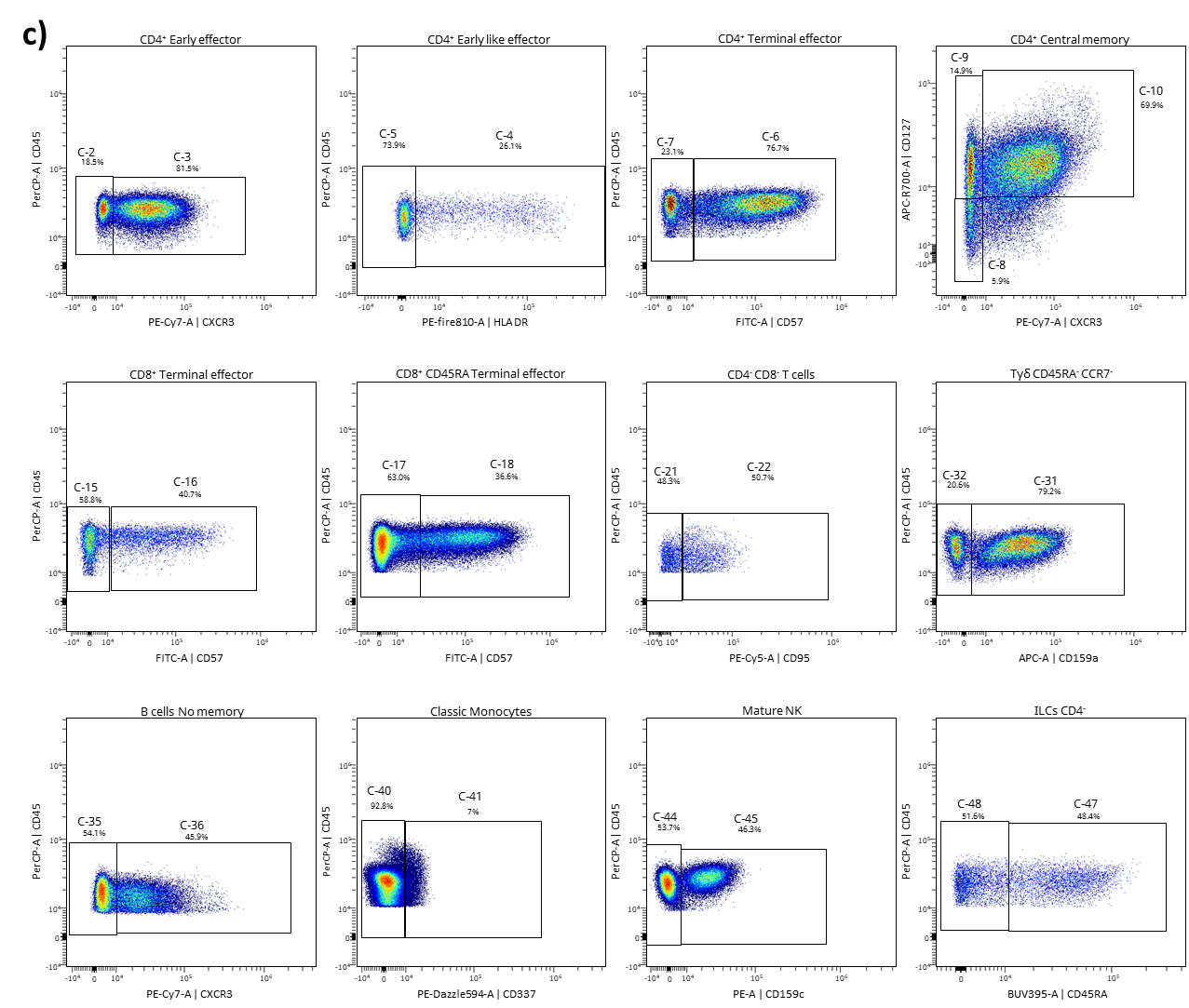


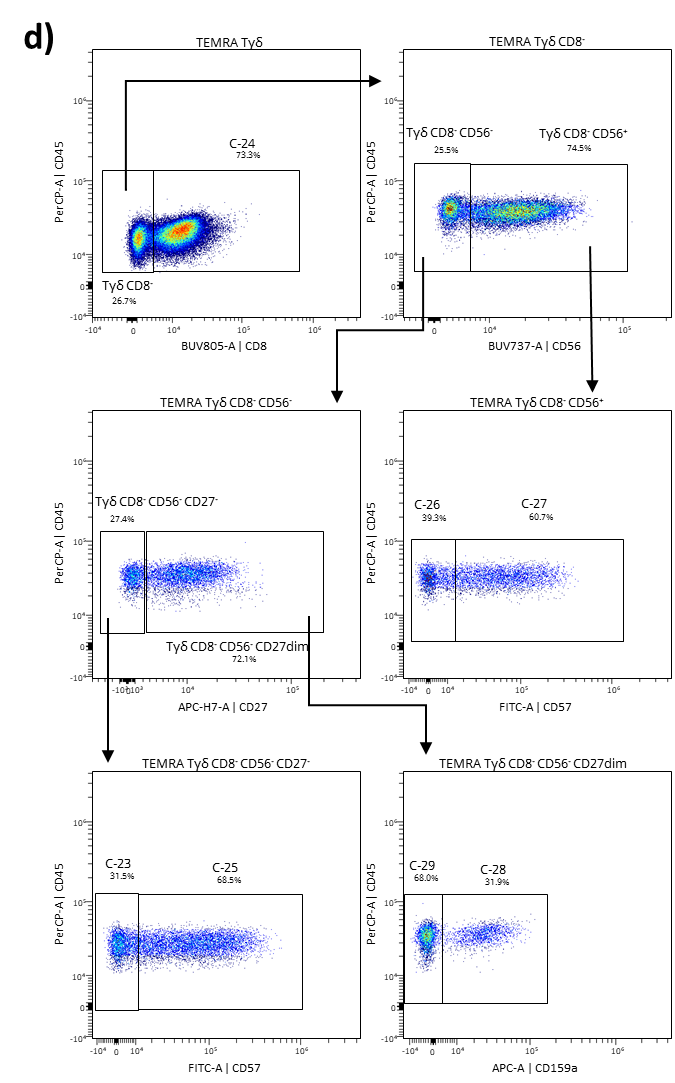


**Supplementary Fig. 1. Hierarchical cell subset identification.**

**(a)** Representative gating strategy used to identify the main cellular populations within total peripheral blood mononuclear cells (PBMC). Arrows are used to visualise the relationships across plots, and numbers are used to call attention to the populations described here. After excluding doublets and dead cells, basophils (1) were delineated as CD45^+^CD123^+^HLA-DR^–^. Dendritic cells (DCs, 2) were identified within CD14^−^CD16^−^ as CD3^–^CD19^–^CD123^+^HLA-DR^+^ (plasmacytoid DCs [pDCs]) and CD3^–^CD19^–^CD11c^+^HLA-DR^+^ (classical or conventional DCs [cDCs]). cDCs were further divided into type 1 (CD141^+^, cDC1) and type 2 (CD1c^+^, cDC2). Monocytes were gated based on FSC-A/SSC-A properties and further classified into non-classical (CD14^−^CD16^+^), intermediate (CD14^+^CD16^+/low^) and classical (CD14^+^CD16^–^). From the lymphogate, B-cells (4) were gated out of CD3^−^TCRγδ^−^ as CD19^+^ and/or CD20^+^ cells. B-cells were further gated as IgD^+^CD27^−^, IgD^+^CD27^+^ or IgD^−^CD27^+/−^; the IgD^−^CD27^+/−^ subset was divided into plasmablasts or IgD^−^ memory B-cells based on CD20 and CD27 expression. Natural killer (NK) cells (5) were defined within the lymphogate as CD3^−^TCRγδ^−^HLA-DR^−^ and classified as early NK (CD56^+^CD16^−^), mature NK (CD56^+^CD16^+^) and terminal NK (CD56^−^CD16^+^) cells. Innate lymphoid cells (ILCs, 6) were gated within the lymphogate as CD3^−^CD19^−^CD20^−^CD14^−^CD123^−^CD127^+^CD2^+^ and further divided into subsets based on the expression of CD4. **(b)** T-cells were identified within the lymphogate based on the expression of CD3. Total Tγδ cells (7) were identified as CD3^+^TCRγδ^+^ and divided into subsets based on the expression of CD45RA and CCR7. Total NKT-like cells (8) were identified in the CD3^+^TCRγδ^-^ compartment as CD56^+^. The inclusion of CD2 and CD8 allowed further classification of NKT-like cells. Real T-cells were defined as CD3^+^TCRγδ^–^CD56^–^ and further divided into CD4^+^, CD8^+^, CD4^+^CD8^+^ and CD4^−^CD8^−^ T-cells. Regulatory T-cells (T_regs_, 9) were identified within total CD4^+^ T-cells as CD127^low/–^CD25^high^. CD39 and CD45RA were used to classify them further. Within total CD4^+^ T-cells (10) and CD8^+^ T-cells (11), CCR7, CD45RA, CD27 and CD28 were used to divide them into different T-cell phenotypes as shown in the figure. **(c)** Hierarchical gating was also used to identify the different clusters identified in Fig. 1 and Table 1 within the subset described on the top of each plot. CD4 early effector cells were classified by CXCR3 expression as CD4 early effector (1) (CXCR3^−^) and CD4 early effector (2) (CXCR3^+^). CD4 early-like effector cells were classified by HLA-DR expression as CD4 early-like effector (1) (HLA-DR^+^) and CD4 early-like effector (2) (HLA-DR^−^). CD4 terminal effector cells were classified by CD57 expression as CD4 terminal effector (1) (CD57^+^) and CD4 terminal effector (2) (CD57^−^). CD4 central memory cells were divided into CD127^+^CXCR3^+^ (CD4 central memory [3]), CD127^+^CXCR3^-^ (CD4 central memory [2]) and CD127^-^CXCR3^-^ (CD4 central memory [1]). CD8^+^ terminal effector cells was classified by CD57 expression as CD8^+^ terminal effector (1) (CD57^−^) and CD8^+^ terminal effector (2) (CD57^+^). CD8^+^CD45RA^+^ terminal effector cells were classified by CD57 expression as CD8^+^CD45RA^+^ terminal effector (1) (CD57^−^) and CD8^+^CD45RA^+^ terminal effector (2) (CD57^+^). CD4^–^CD8^–^ T-cells were classified by CD95 expression as CD4^–^CD8^–^ T-cells (1) (CD95^−^) and CD4^–^CD8^–^ T-cells (2) (CD95^+^). CD45RA^–^CCR7^–^ Tγδ cells were classified by CD159a expression as CD45RA^–^CCR7^–^ Tγδ (1) (CD159a^+^) and CD45RA^–^CCR7^–^ Tγδ (2) (CD159a^−^). Non-memory B-cells were classified by CXCR3 expression as non-memory B-cells (1) (CXCR3^−^) and non-memory B-cells (2) (CXCR3^+^). Classical monocytes were classified by CD337 expression as classical monocytes (1) (CD337^−^) and classical monocytes (2) (CD337^+^). Mature NK cells were classified by CD159c expression as mature NK cells (1) (CD159c^−^) and mature NK cells (2) (CD159c^+^). CD4^–^ ILCs were classified by CD45RA expression as CD4^–^ ILCs (1) (CD45RA^+^) and CD4^–^ ILCs (2) (CD45RA^−^). **d)** Given the large number of identified TEMRA Tγδ cell clusters, the gating strategy to identify them is shown. This population was divided into CD8^+^ (Tγδ CD45RA^+^CCR7^–^ [2]) and CD8^–^ subsets. The inclusion of CD56 enables further classification of TEMRA Tγδ cells. Hence, CD45RA^+^CCR7^–^CD8^–^CD56^+^ Tγδ cells were then divided by CD57 expression as CD45RA^+^CCR7^–^ Tγδ (4) (CD57^–^) and CD45RA^+^CCR7^–^ Tγδ (5) (CD57^+^). CD27 expression was used to classify CD45RA^+^CCR7^–^CD8^–^CD56^–^ Tγδ cells. Then, CD45RA^+^CCR7^–^CD8^–^CD56^–^CD27^–^ Tγδ were classified by CD57 expression as CD45RA^+^CCR7^–^ Tγδ (1) (CD57^–^) and CD45RA^+^CCR7^–^ Tγδ (3) (CD57^+^). Finally, CD159a was used to divide CD45RA^+^CCR7^–^CD8^–^CD56^–^ CD27^dim^ Tγδ cells into CD45RA^+^CCR7^–^ Tγδ (6) (CD159a^+^) and CD45RA^+^CCR7^–^ Tγδ (7) (CD159a^–^).


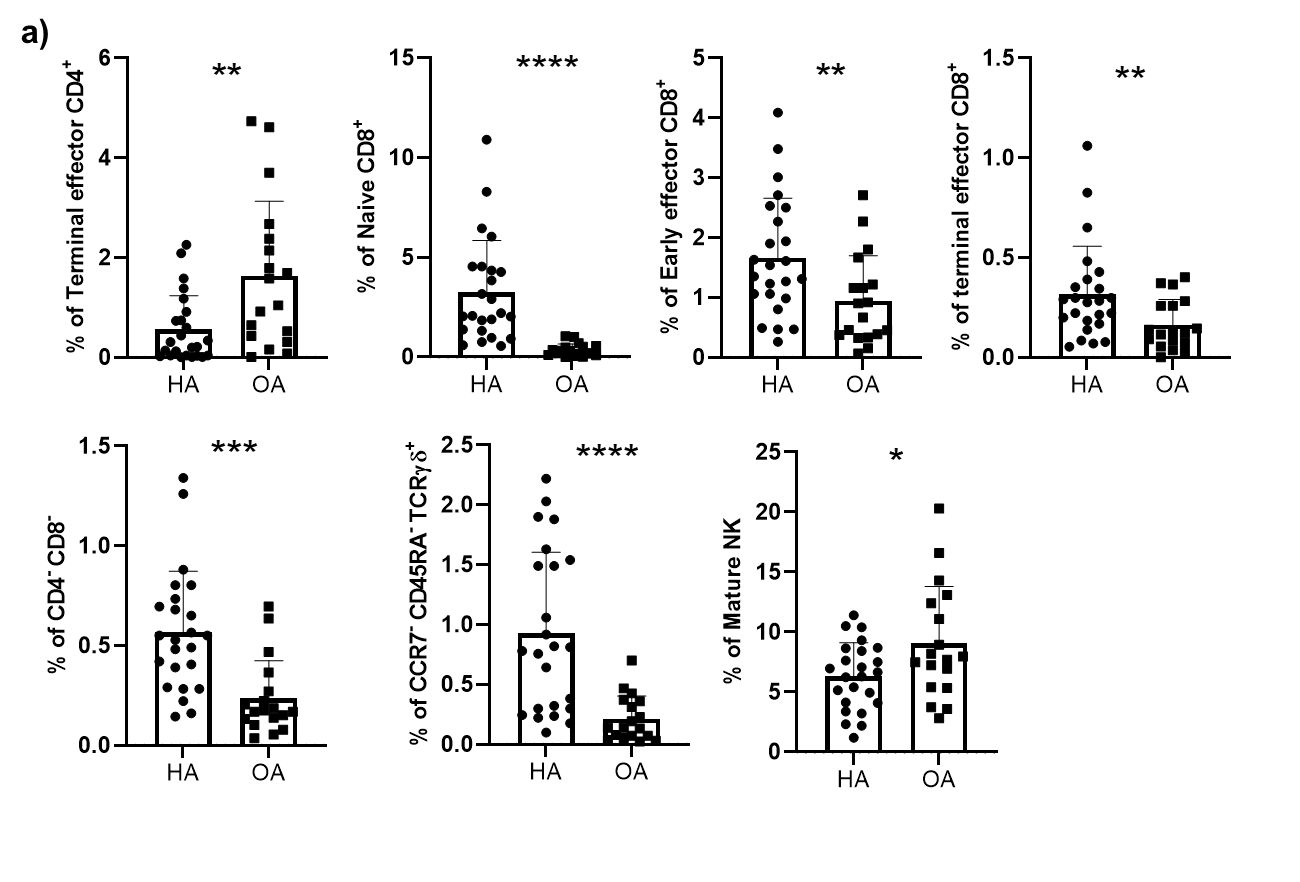

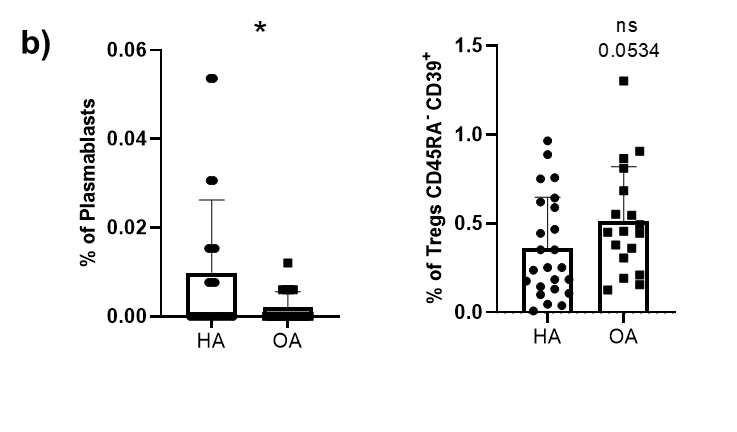

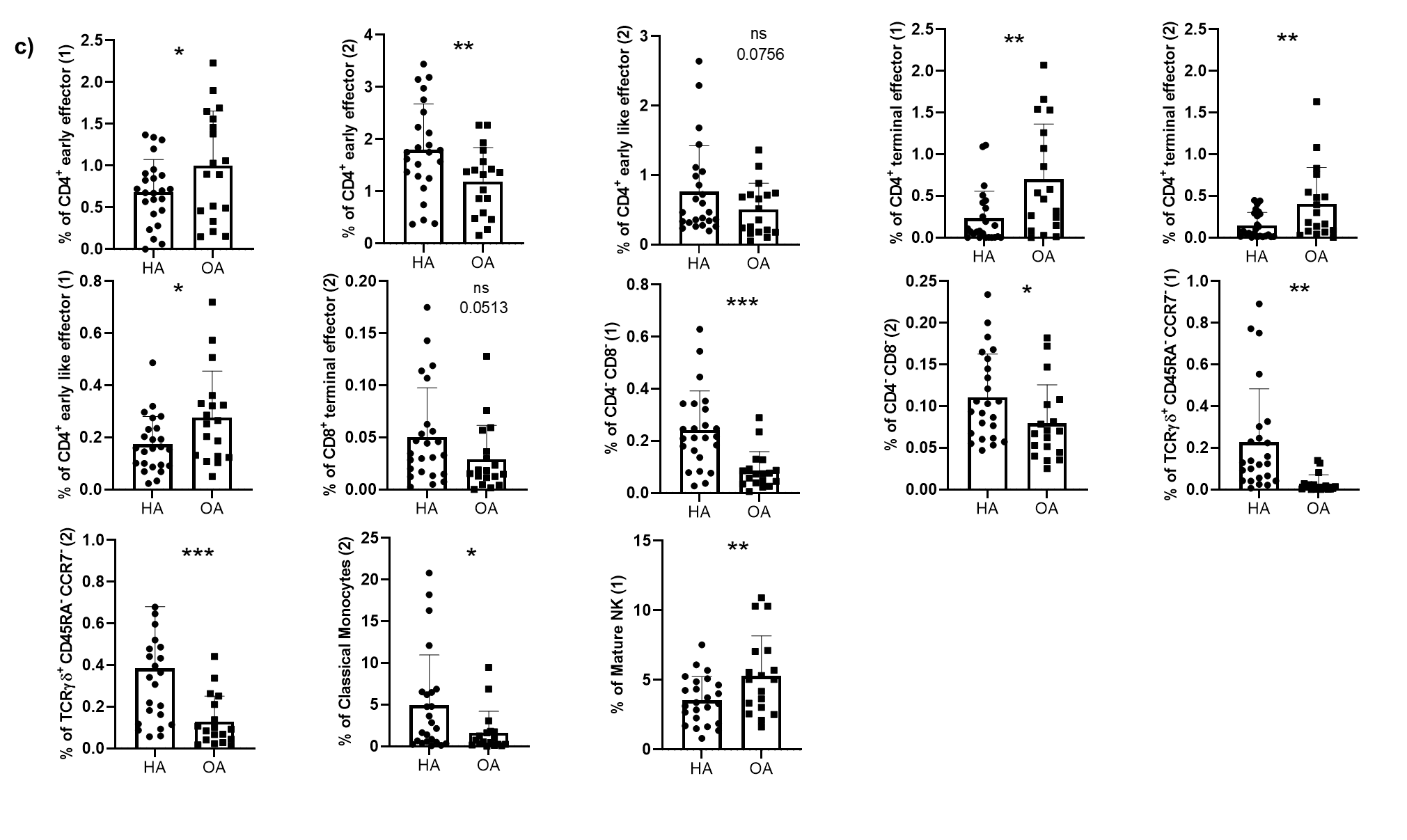

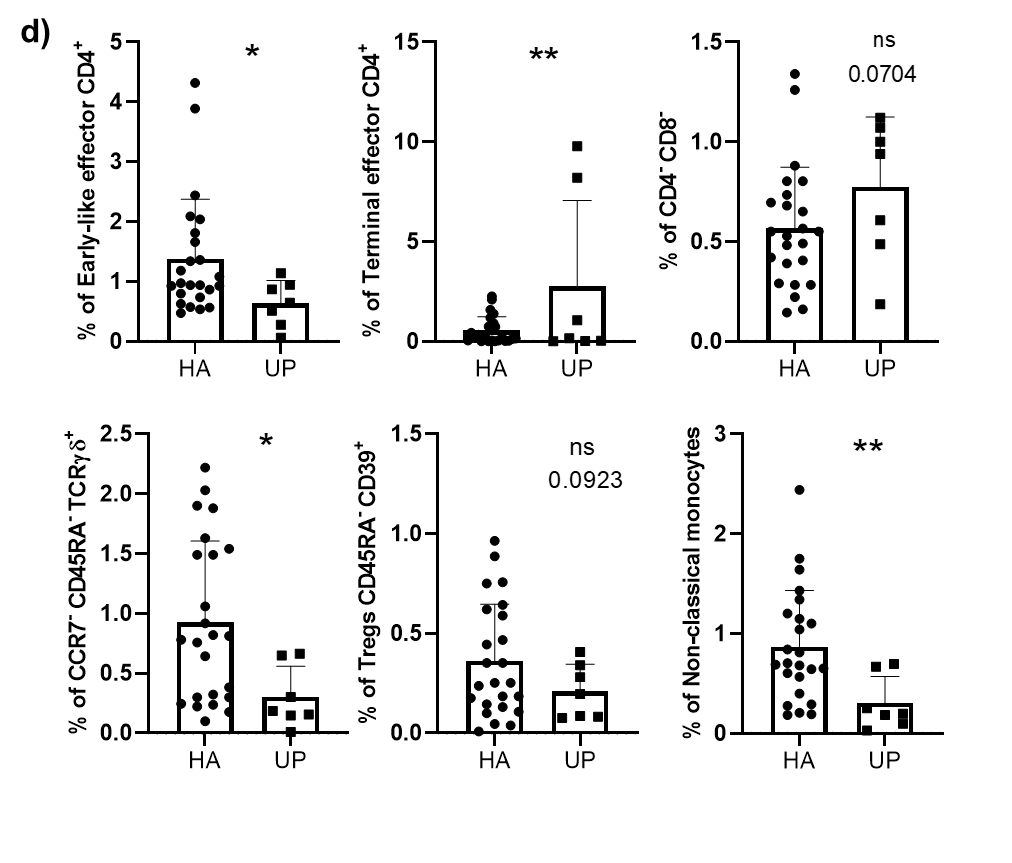

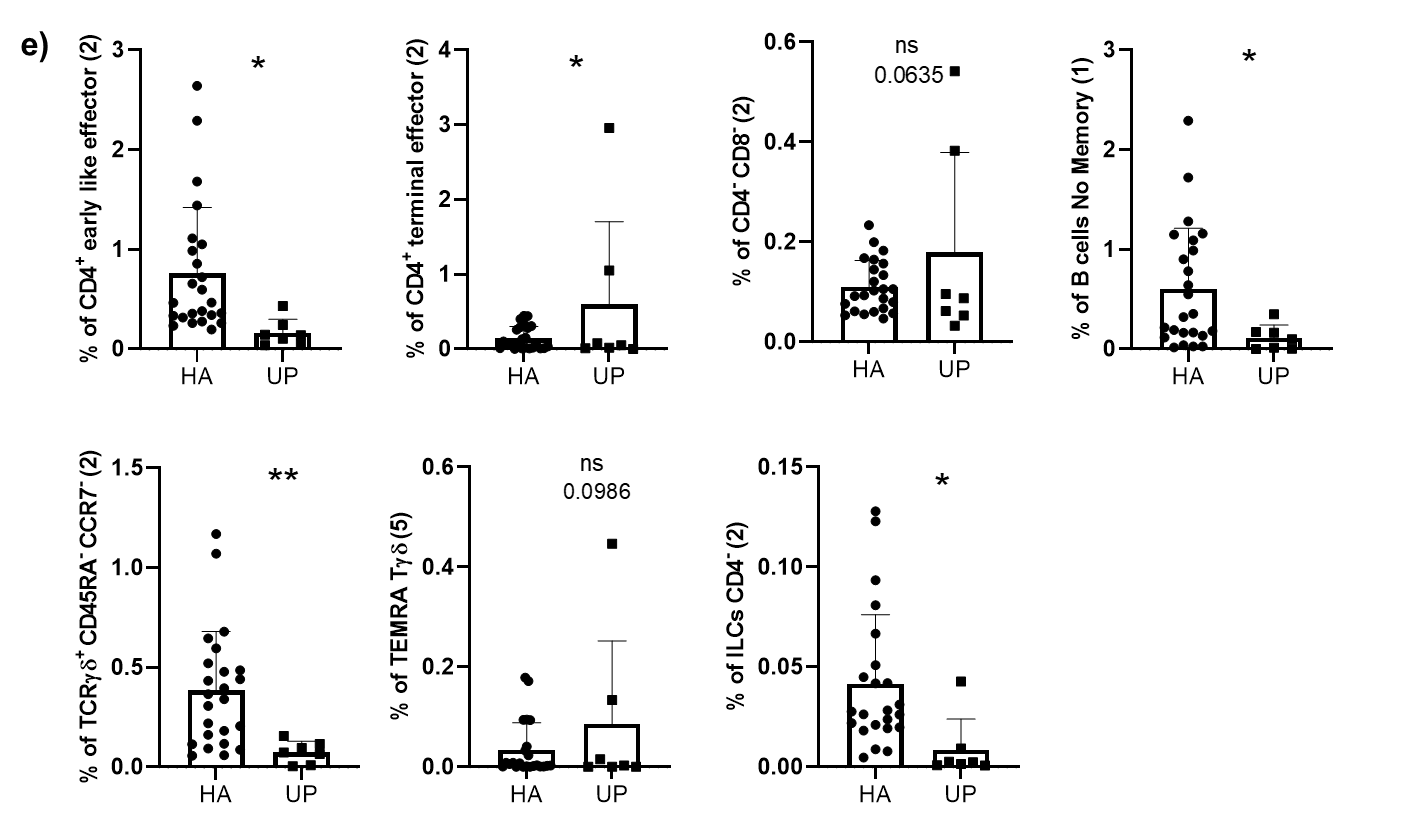


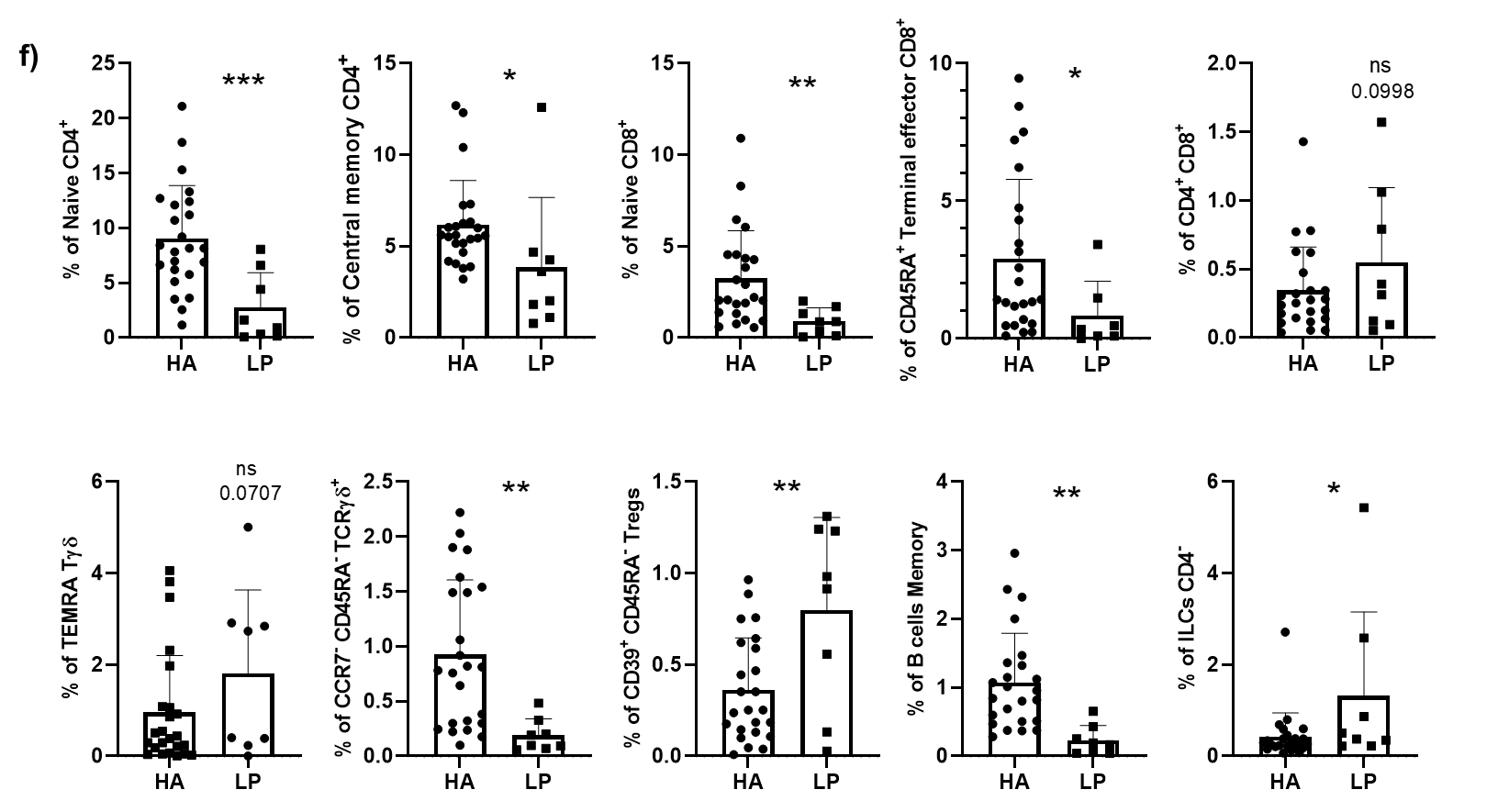


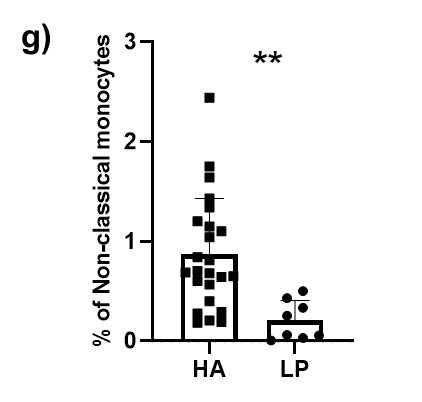

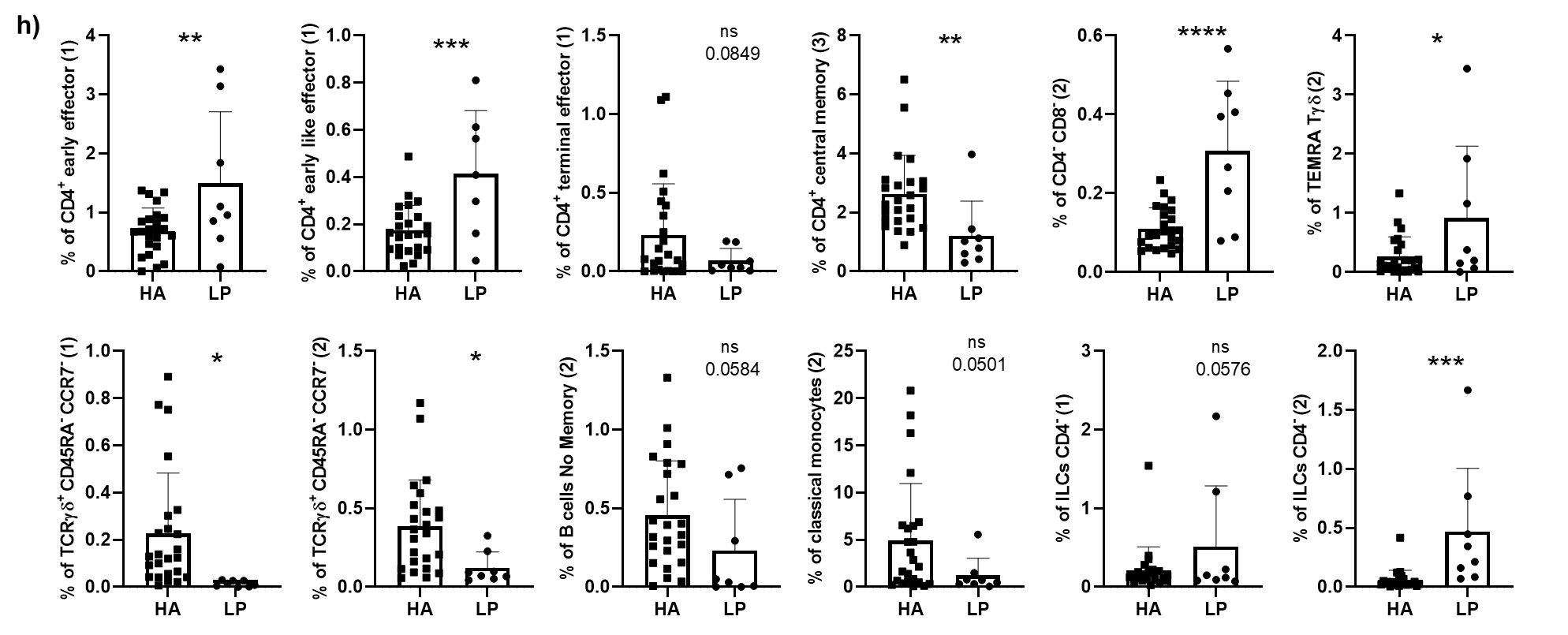

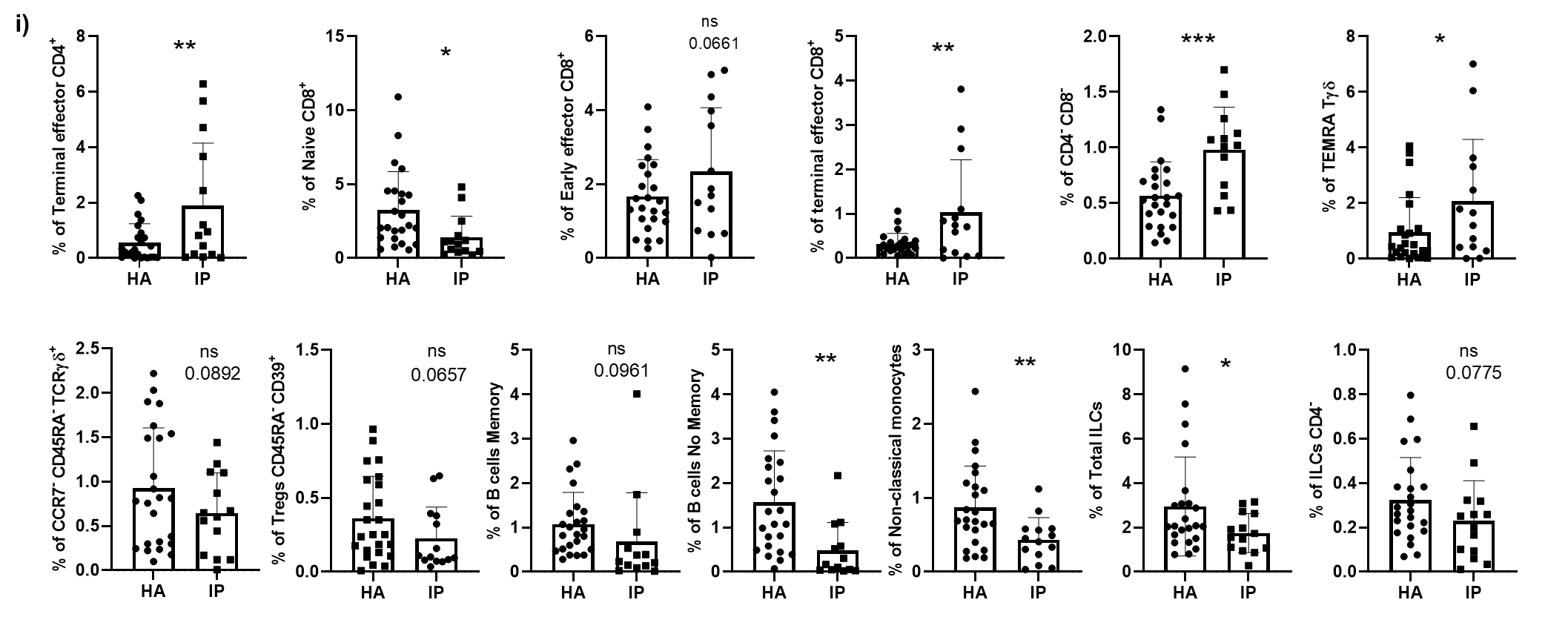

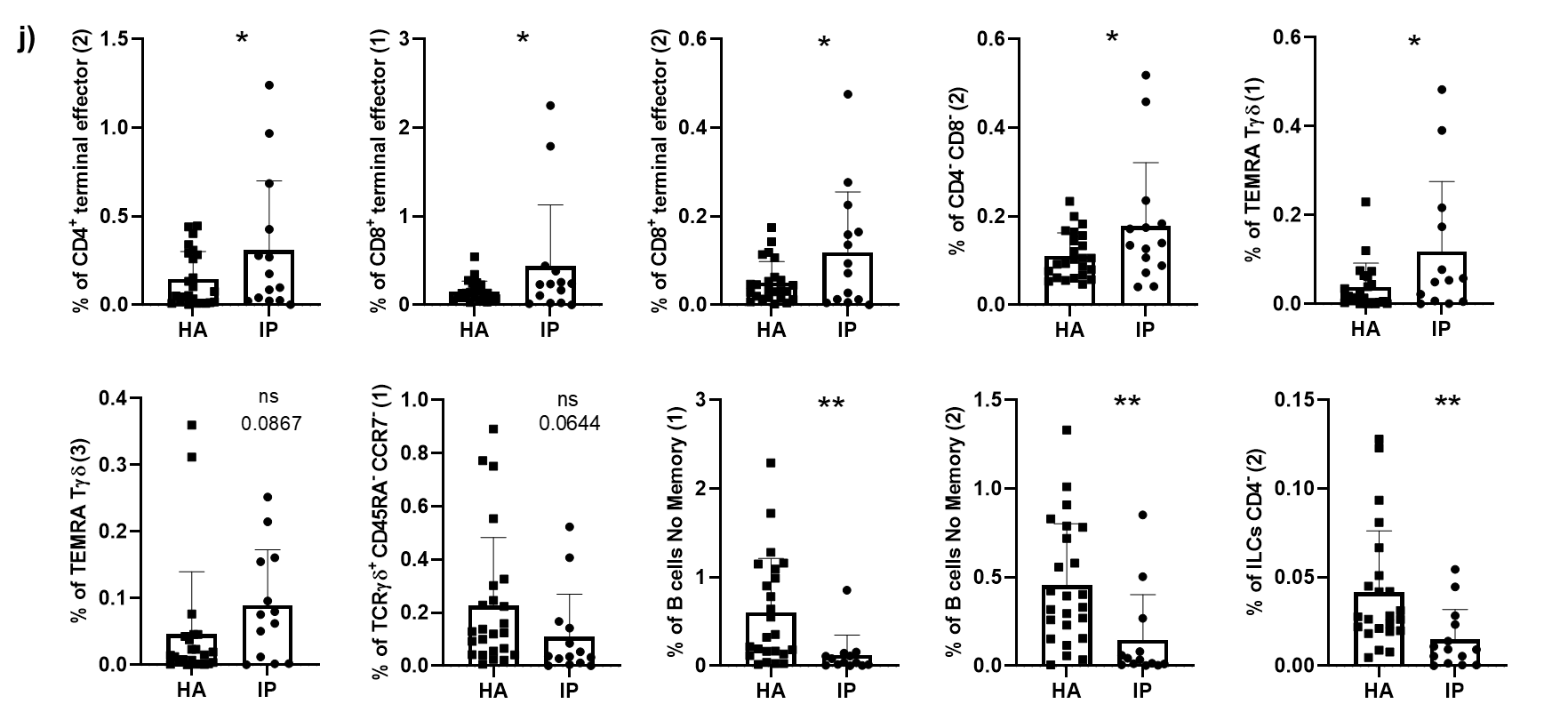

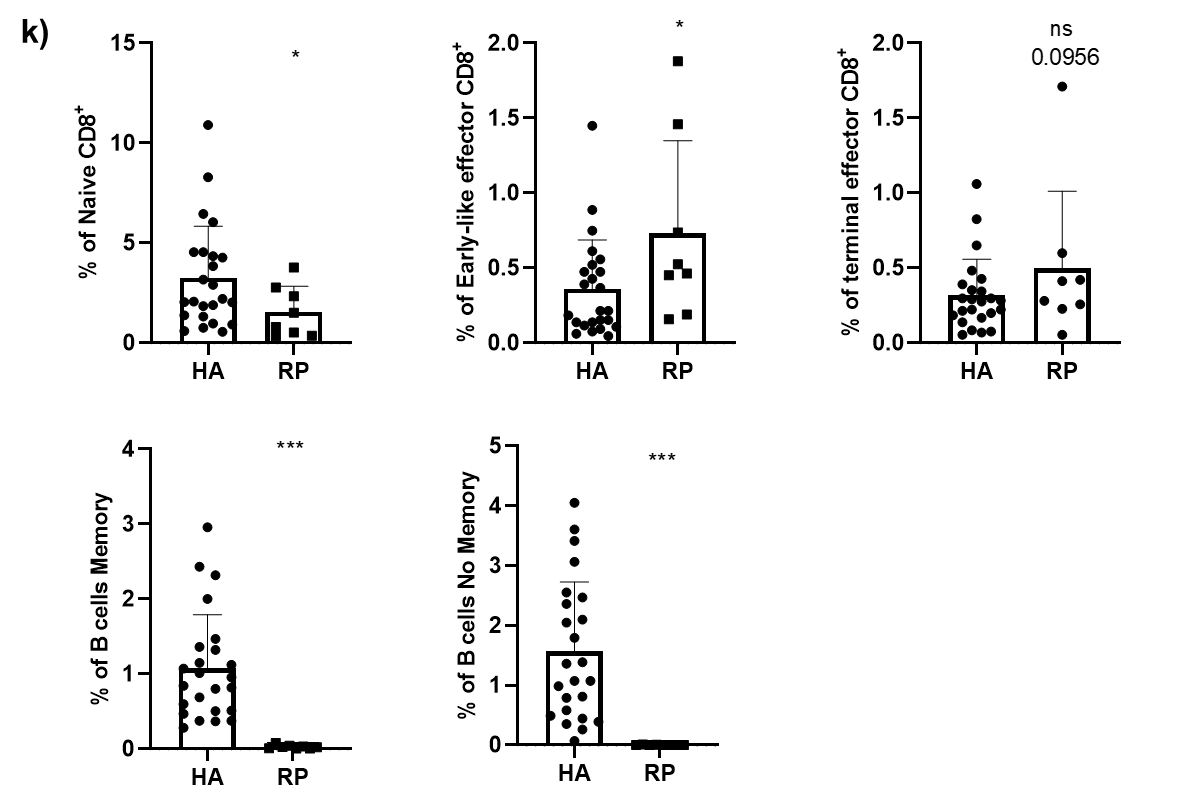

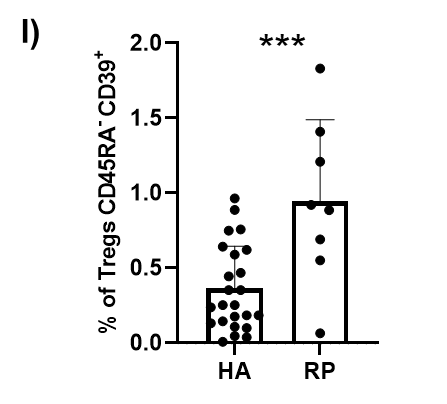

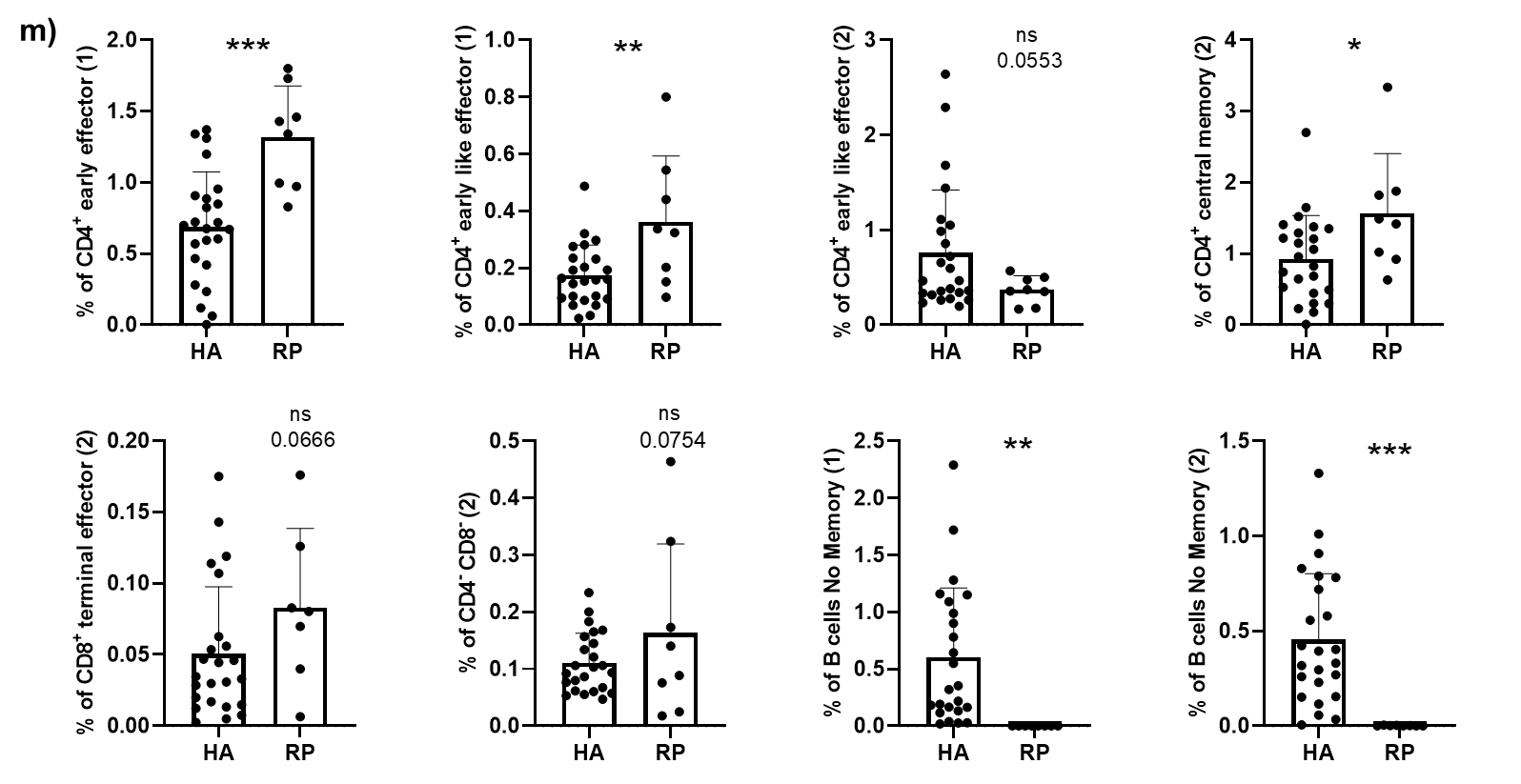


**Supplementary Fig. 2. In-depth analysis of the different cohorts at baseline.**

**(a)** Validation by hierarchical gating, as shown in Supplementary Fig. 1, of the cell populations differentially expressed between healthy adults (HA) and older adults (OA) in the volcano plot from Fig. 2c. Those samples that could not be analysed by the volcano plots due to the low number of events are shown in **(b).** Analysis of the specific clusters within a given population is shown in **(c)**. **(d)** Comparison by hierarchical gating of the populations differentially expressed between HA and untreated oncohaematologic patients (UP) as revealed in Fig. 2d. The specific cluster comparison is shown in **(e)**. **(f)** Study of the cell populations differentially expressed between HA and lenalidomide-treated oncohaematologic patients (LP) as shown in Fig. 2e. Those samples that could not be analysed by the volcano plots due to the low number of events are shown in **(g)**. Analysis of the specific clusters within a given population is shown in **(h)**. **(i)** Comparison by hierarchical gating of the cell populations differentially expressed between HA and ibrutinib-treated oncohaematologic patients (IP) as shown in Fig. 2f. Analysis of the specific clusters within a given population is shown in **(j)**. **(k)** Validation by hierarchical gating of the cell populations differentially expressed between HA and rituximab-treated oncohaematologic patients (RP) as shown in Fig. 2g. Those samples that could not be analysed by the volcano plots due to the low number of events are shown in **(l)**. Analysis of the specific clusters within a given population is shown in **(m)**. In all cases, a t-test was performed where p < 0.05 was considered significant (*p < 0.05; **p < 0.01; ***p < 0.001). A p-value between 0.05 and 0.1 was considered not significant (ns) but with a relevant trend (the exact p-value is shown).


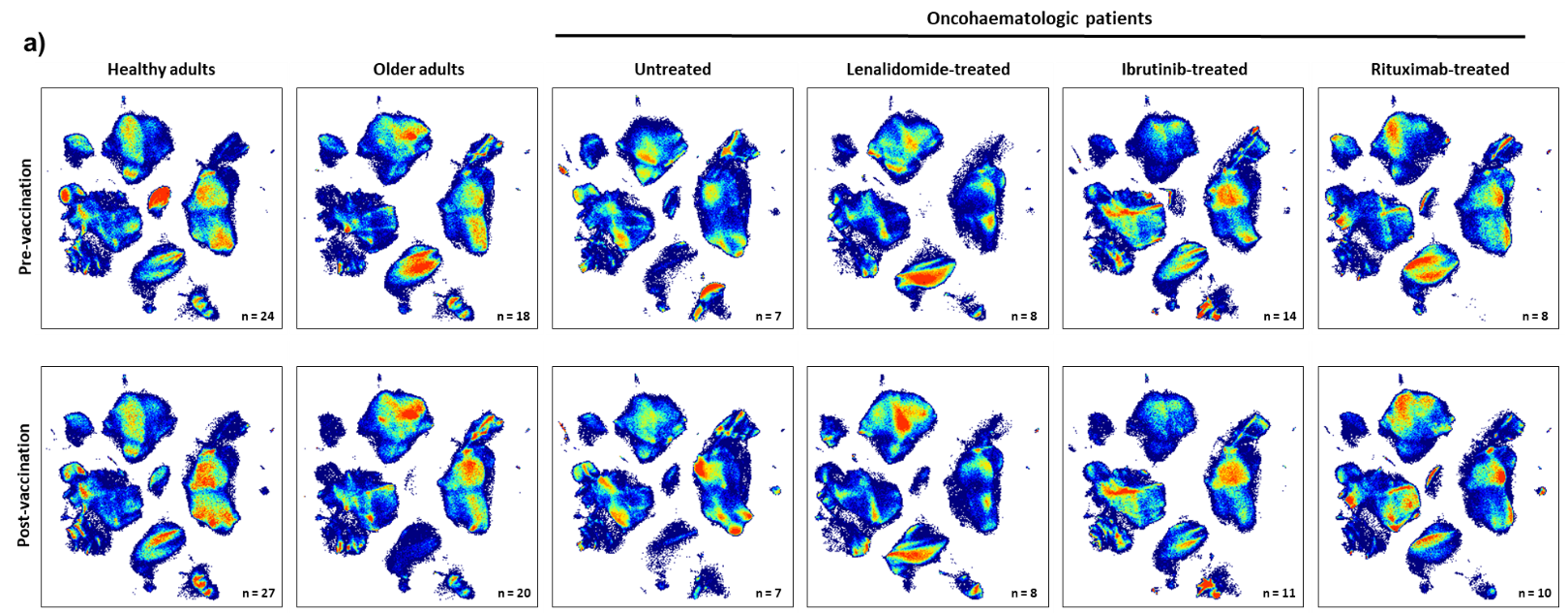


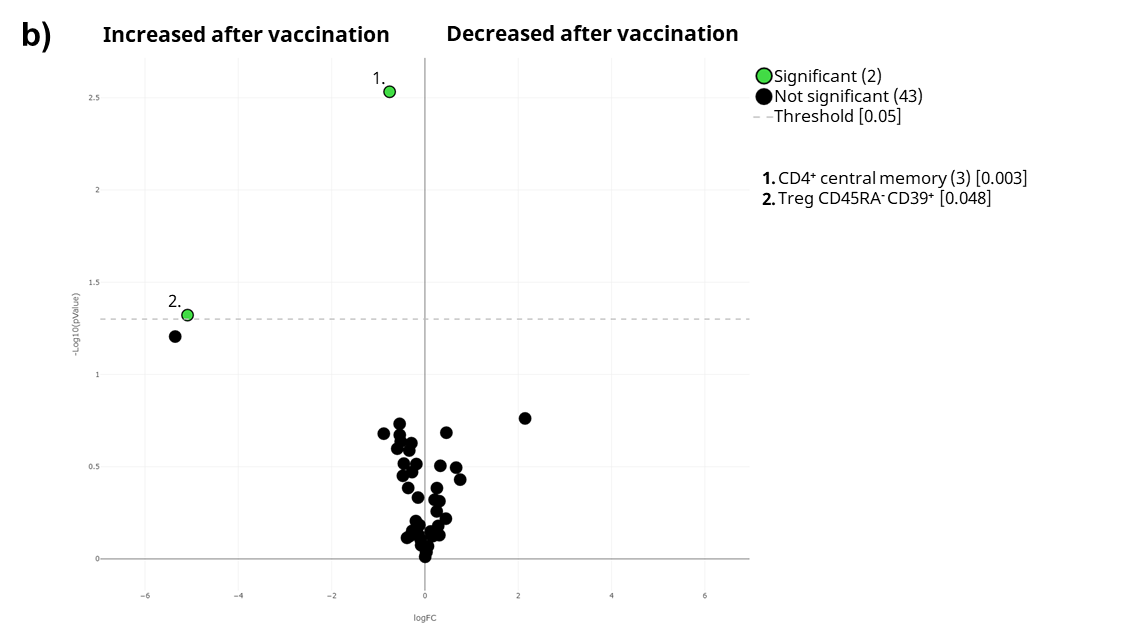

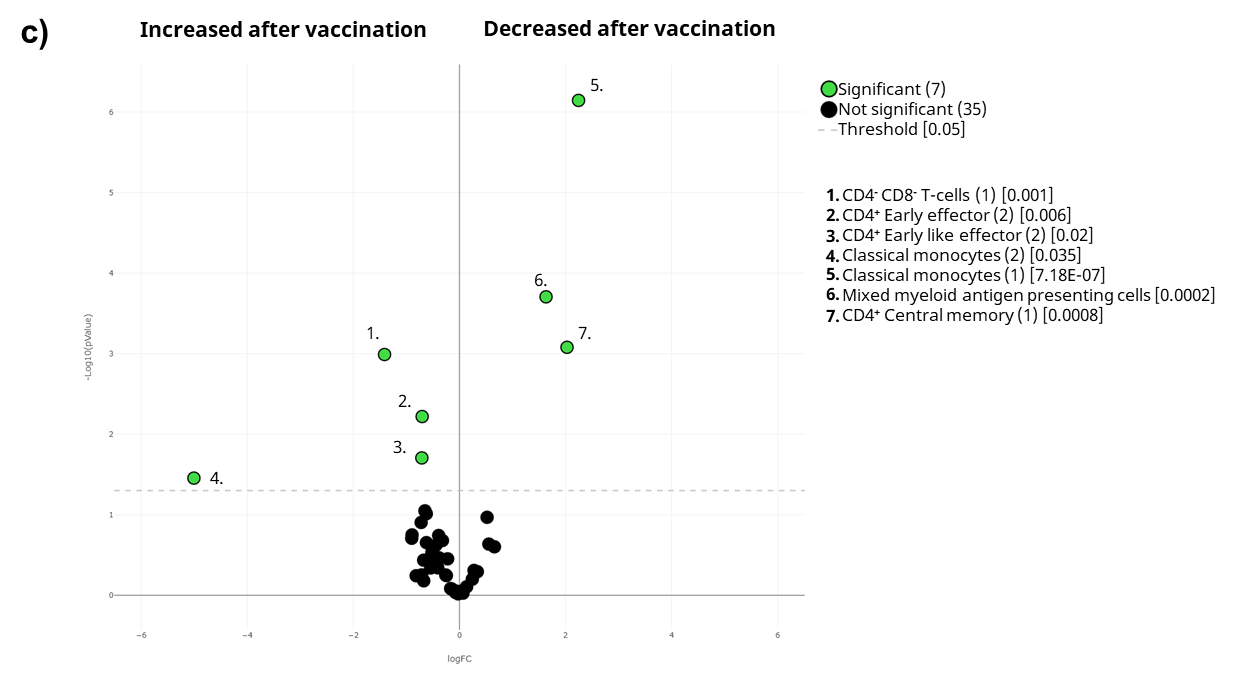

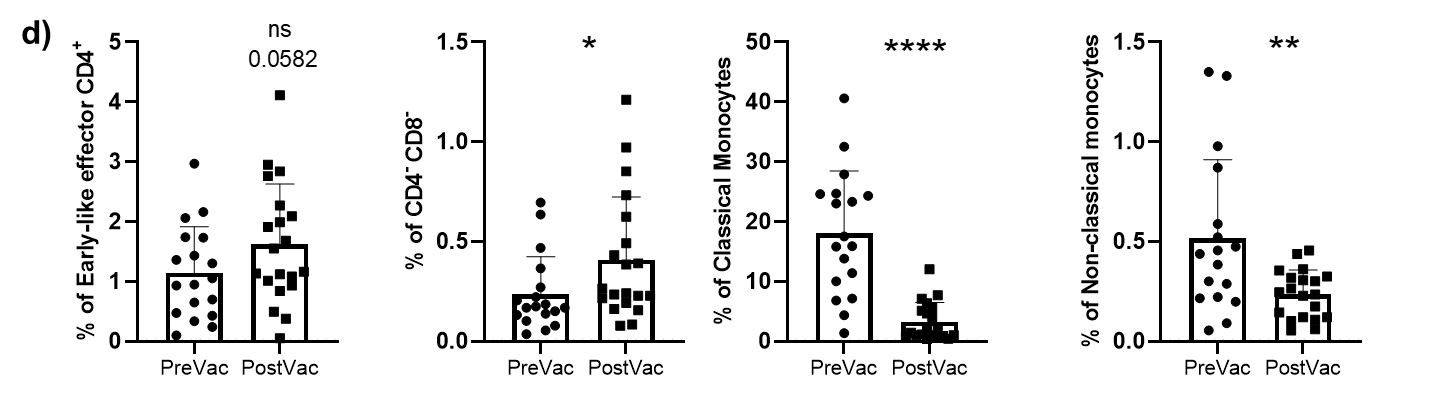


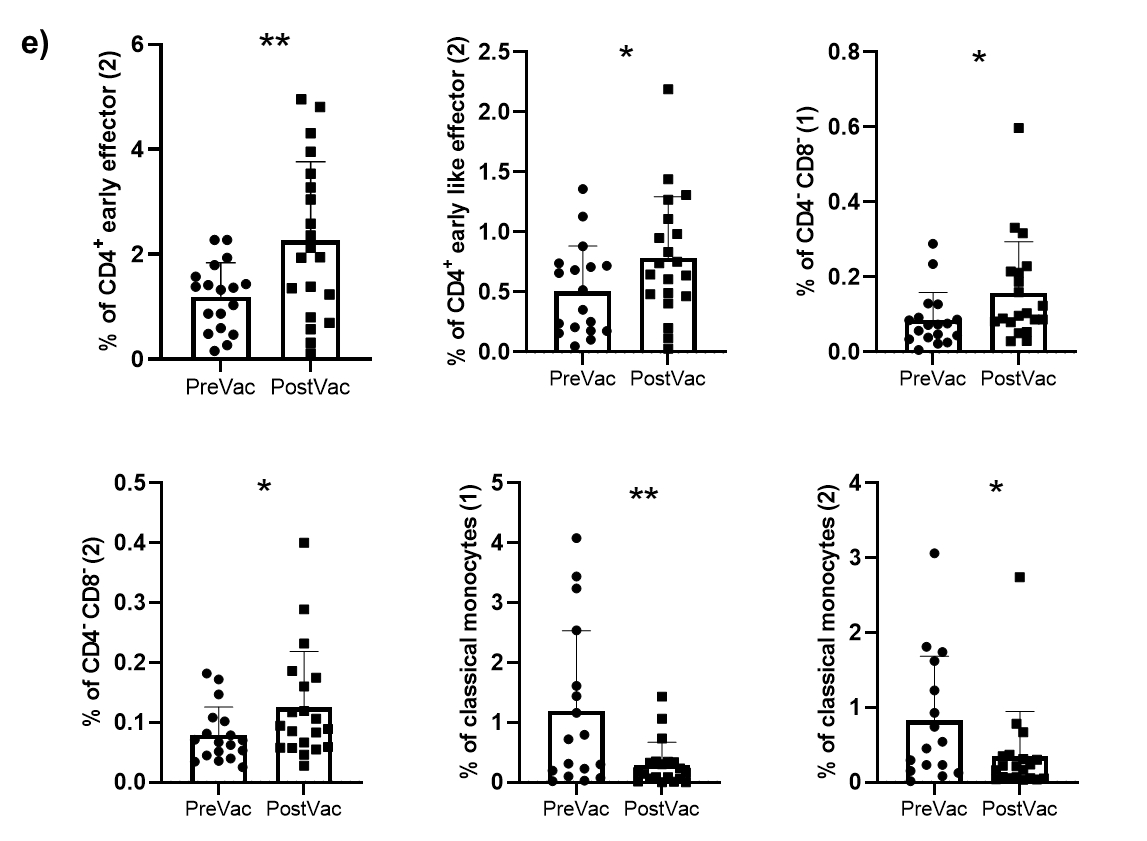


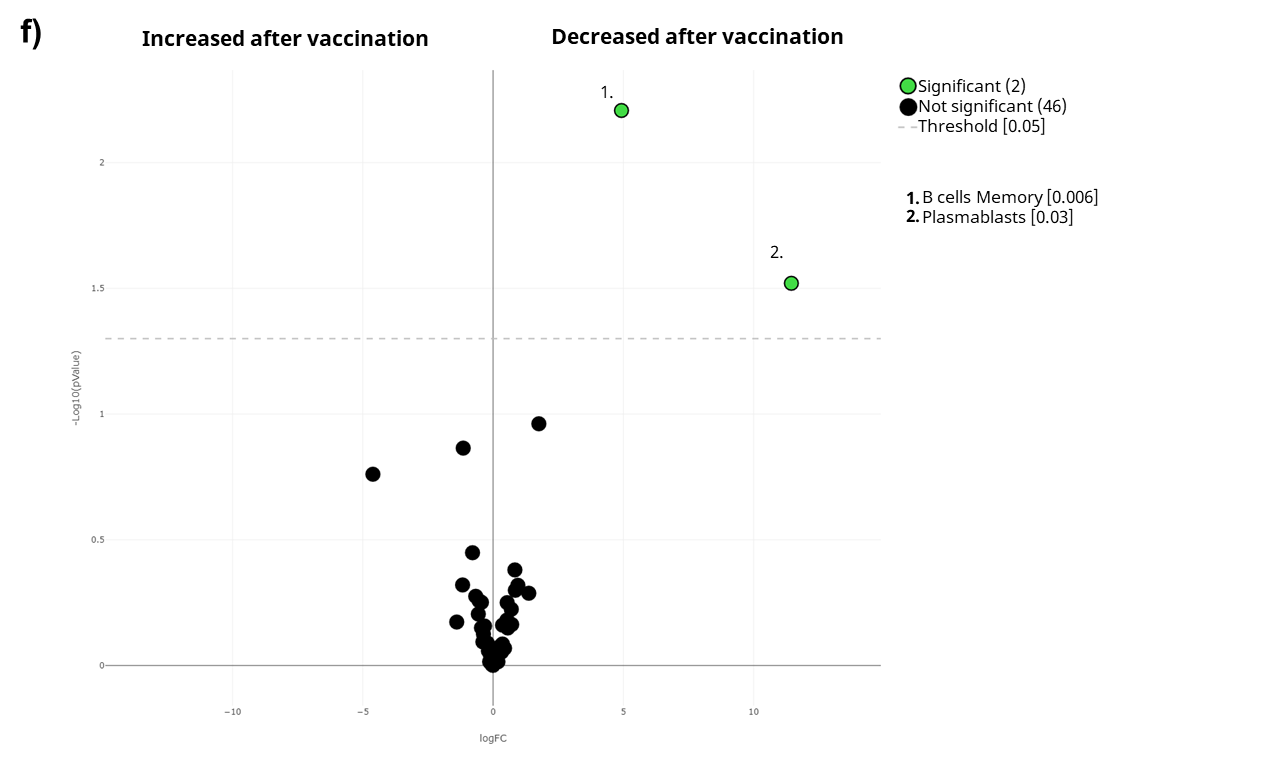


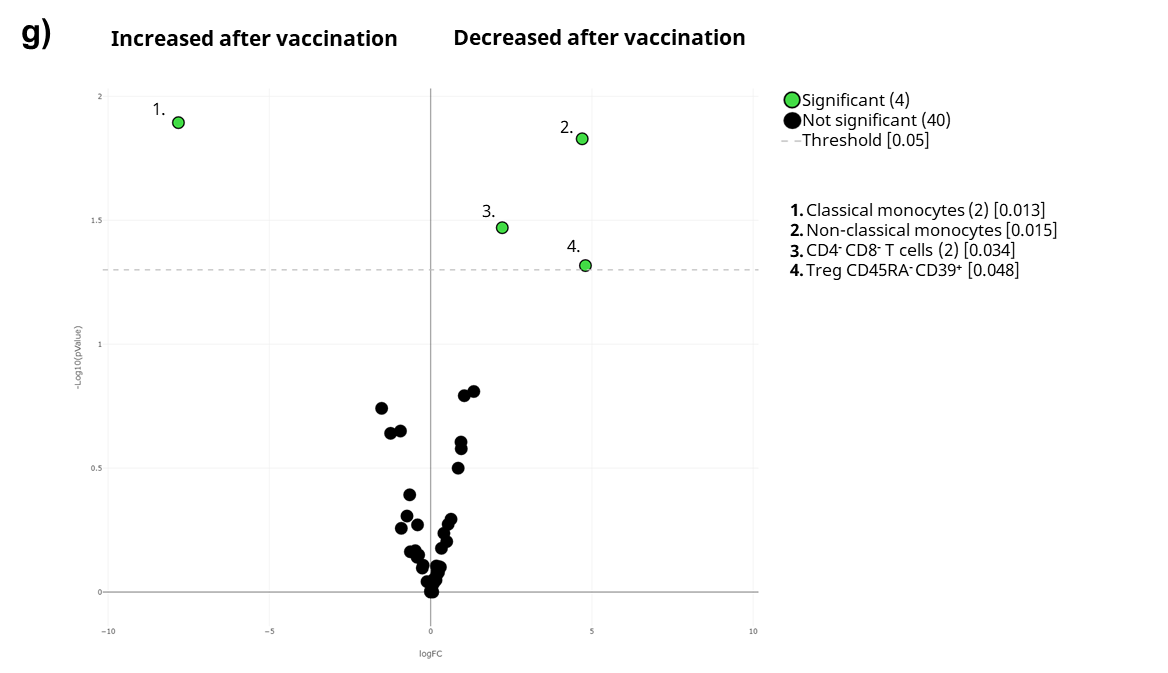


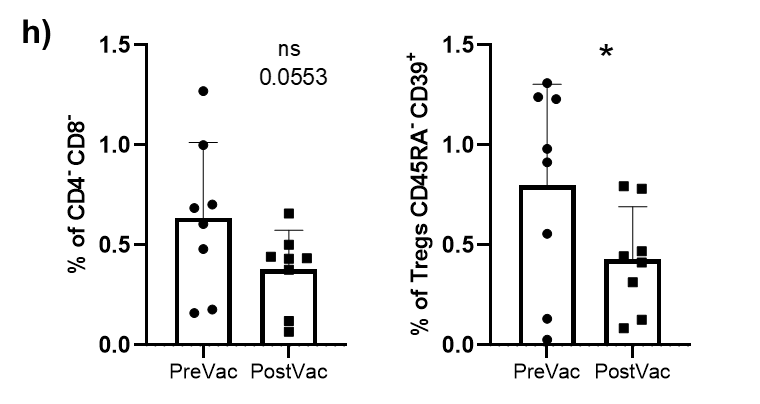


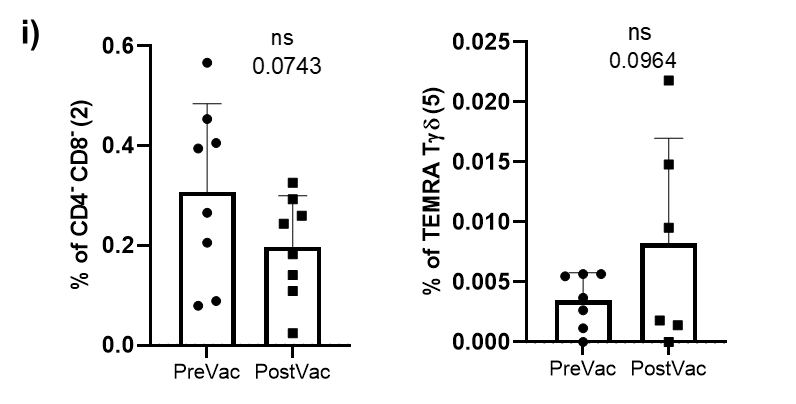


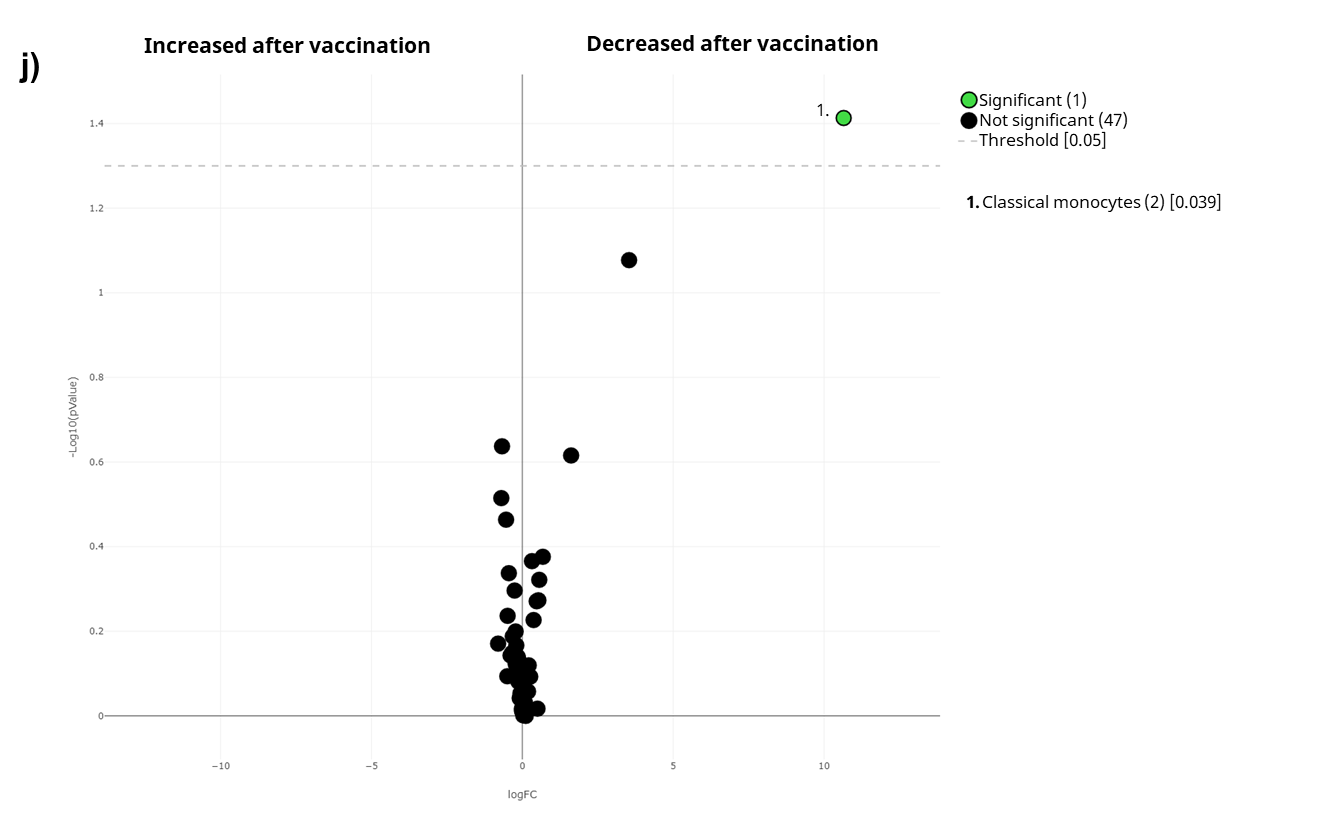


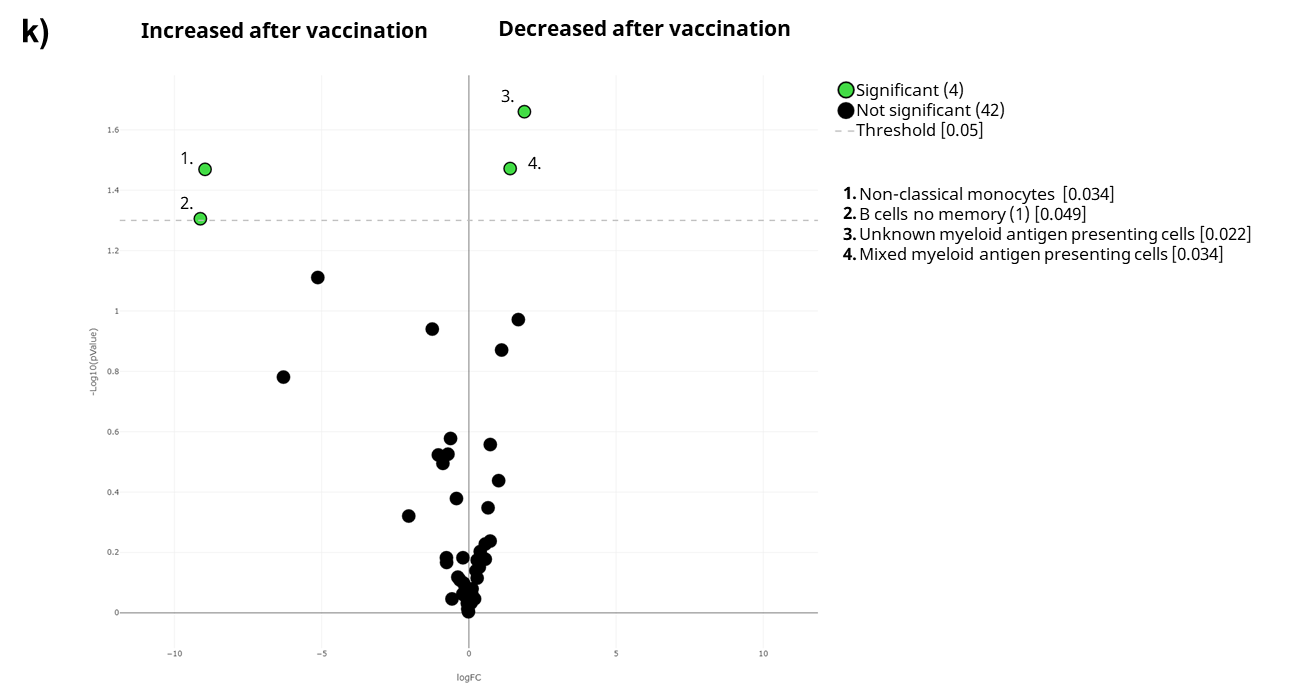

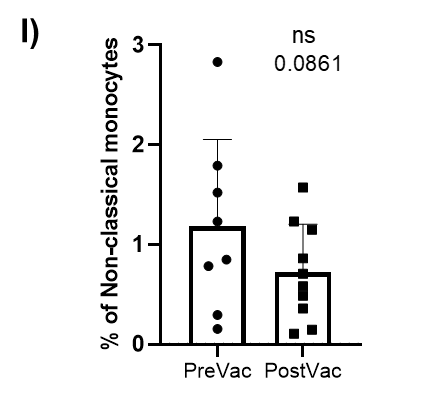

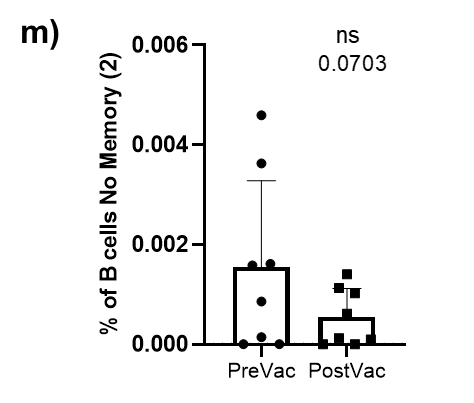


**Supplementary Fig. 3. In-depth immune characterisation of the cohorts before and after vaccination.**

**(a)** UMAP density plots of the cohorts before (above) and after (below) vaccination, including healthy adults (n = 24 and n = 27, respectively), older adults (n = 18 and n = 20, respectively), untreated oncohaematologic patients (n = 7 and n = 7, respectively), lenalidomide-treated oncohaematologic patients (n = 8 and n = 8, respectively), ibrutinib-treated oncohaematologic patients (n = 14 and n = 11, respectively) and rituximab-treated oncohaematologic patients (n = 8 and n = 10, respectively). **(b)** Volcano plot analysis comparing healthy adults before and after vaccination. Clusters that could not be analysed due to a small number of events were plasmablasts, CD4**^–^**/CD8**^–^** T-cells (2) and TEMRA Tγδ cells (4). **(c)** Volcano plot analysis comparing older adults before and after vaccination. Clusters that could not be analysed due to a small number of events were non-classical monocytes, plasmablasts, CD4**^–^**/CD8**^–^** T-cells (2), TEMRA Tγδ cells (4), TEMRA Tγδ (5) and CD45RA**^–^**/CD39**^+^** T_regs_. **(d)** Classical validation, as in Supplementary Fig. 1, of the significantly expressed populations in the volcano plot from **(c)** and **(e)** regarding the specific cell cluster subsets. **(f)** Volcano plot analysis comparing untreated patients before and after vaccination. **(g)** Volcano plot analysis comparing lenalidomide-treated patients before and after vaccination. Clusters that could not be analysed due to a small number of events were plasmablasts, TEMRA Tγδ (4), TEMRA Tγδ (5) and non-memory B-cells (2). **(h)** Classical validation of the significantly expressed clusters in the volcano plot from **(g)**, and **(i)** their specific cell cluster. **(j)** Volcano plot analysis comparing ibrutinib-treated patients before and after vaccination. **(k)** Volcano plot analysis comparing rituximab-treated patients before and after vaccination. Clusters that could not be analysed due to a small number of events were plasmablasts and non-memory B-cells (2). **(l)** Classical validation of the significantly expressed populations in **(k)**. In the volcano plots, green dots represent those clusters that showed significant differences (p < 0.05). A t-test analysis was performed in **(d)**, **(e)**, **(h)**, **(i)**, **(l)** and **(m)**. In all cases, p < 0.05 was considered significant (*p < 0.05; **p < 0.01; ***p < 0.001). A p-value between 0.05 and 0.1 was considered not significant (ns) but with a relevant trend (the exact p-value is shown). ‘PreVac’ and ‘PostVac’ refer respectively to the circulating levels of each cell subset before and after vaccination.


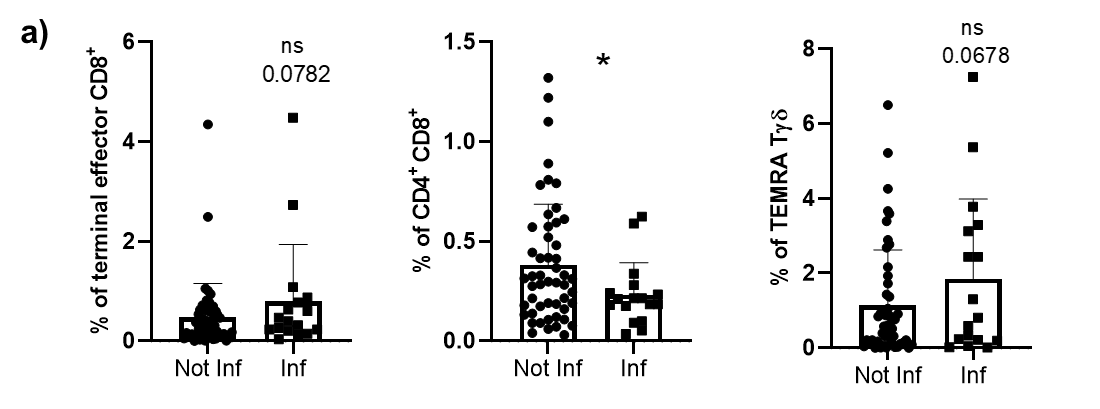


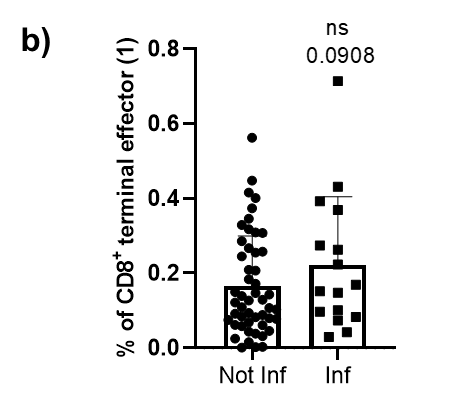

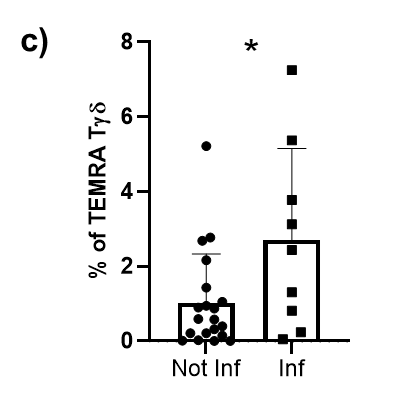

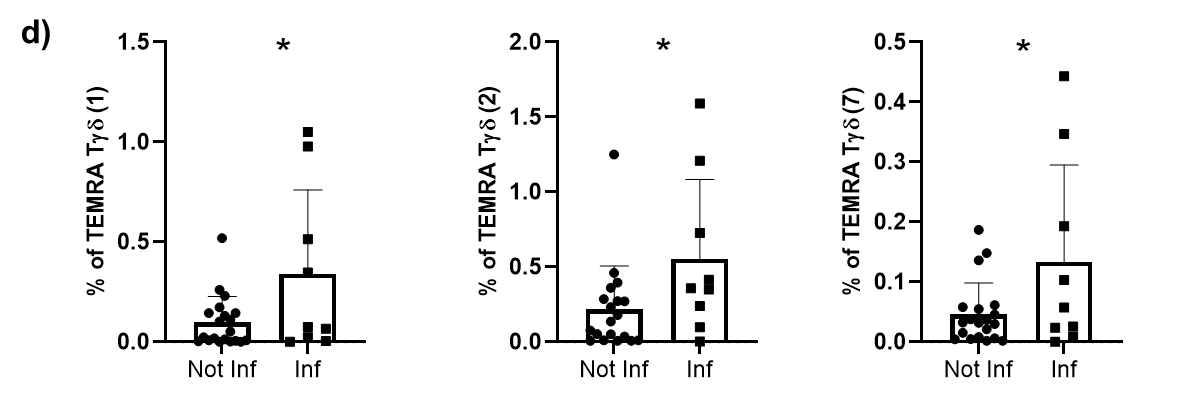


**Supplementary Fig. 4. Immune variations following vaccination predicts SARS-CoV-2 infection.**

**(a)** Classical validation, as in Supplementary Fig. 1, of the significantly expressed populations in the volcano plot from Fig. 3b comparing the immune system of all individuals following immunisation based on their subsequent infection, and **(b)** their specific clusters. **(c)** Hierarchical gating of the significantly expressed populations in the volcano plot from Fig. 3e comparing infected and non-infected oncohaematologic patients, and a subsequent evaluation in **(d)** of the statistically significant cell clusters. In all cases, a t-test was performed where p < 0.05 was considered significant (*p < 0.05). A p-value between 0.05 and 0.1 was considered not significant (ns) but with a relevant trend (the exact p-value is shown). ‘Inf’ refers to subsequently infected individuals, while ‘Not Inf’ are those who were not infected.


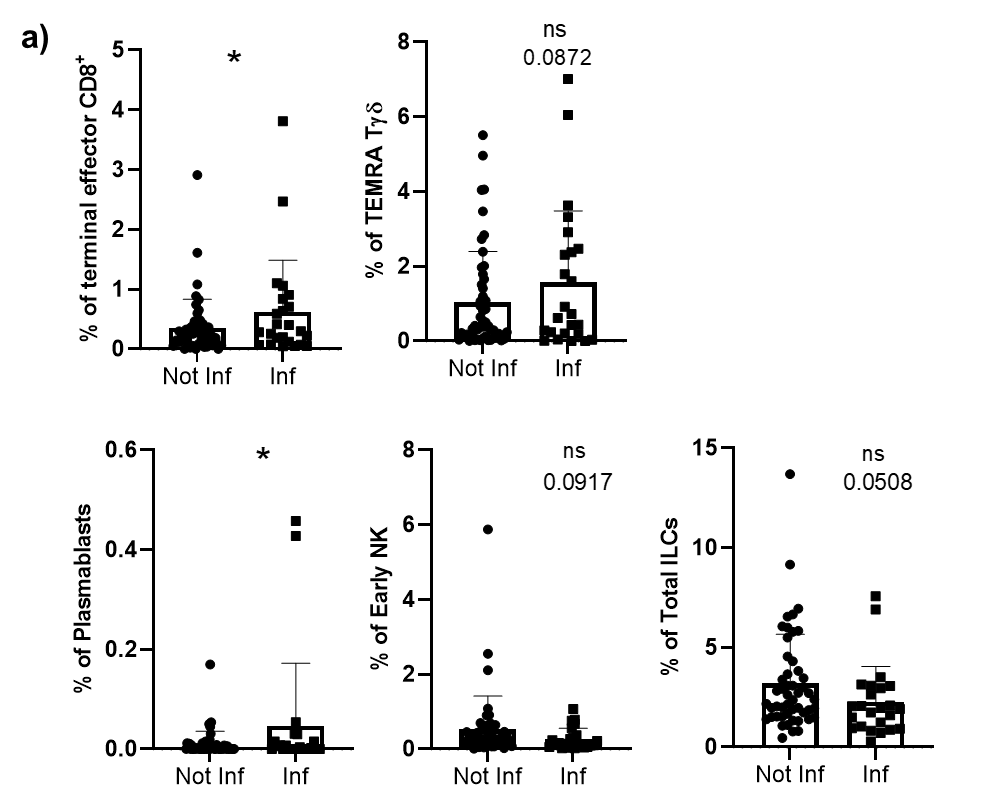

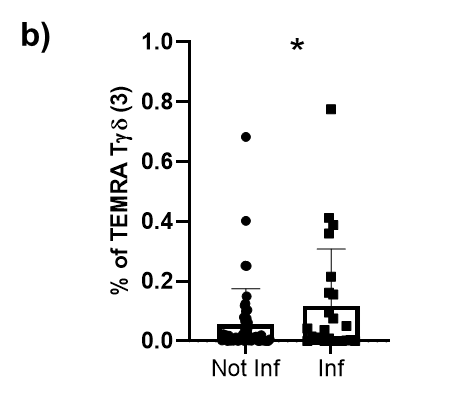


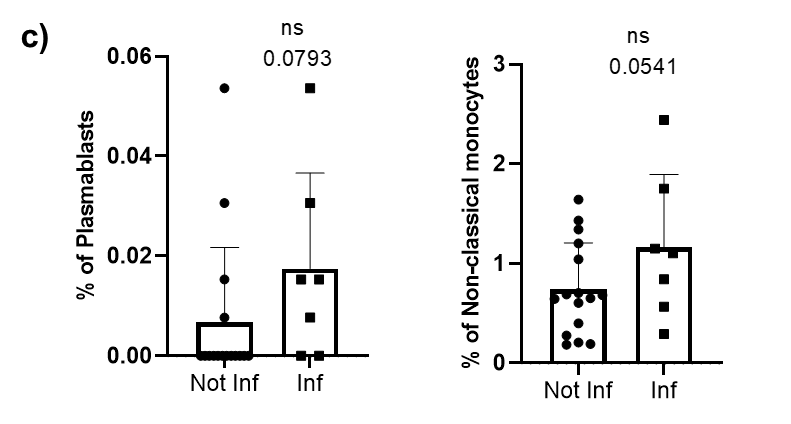


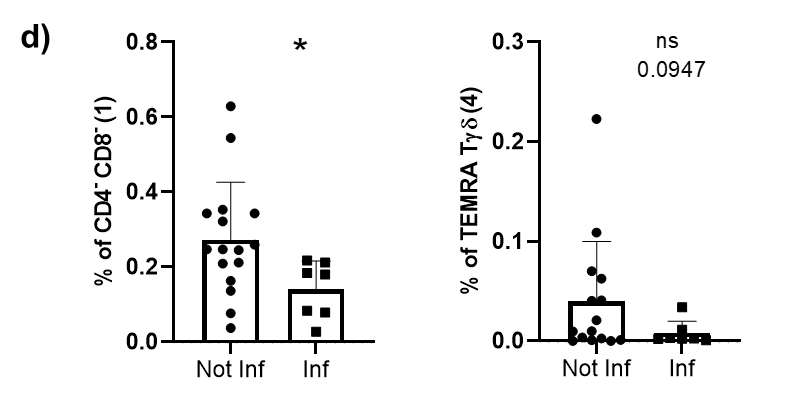

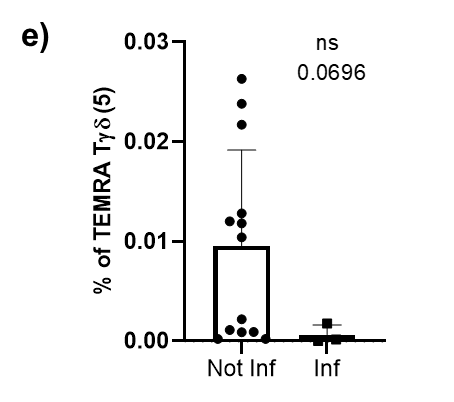


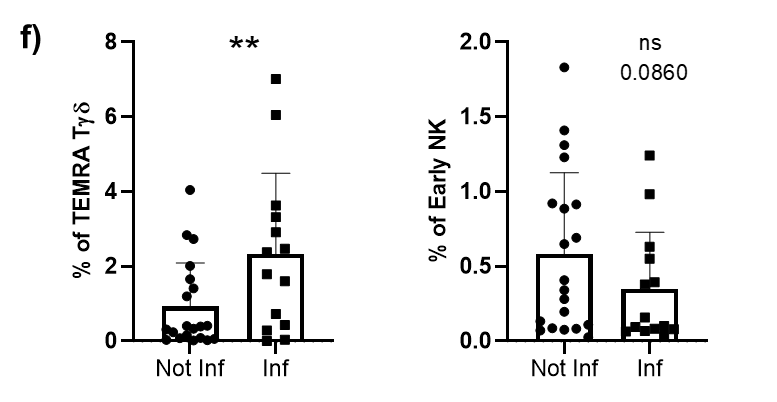

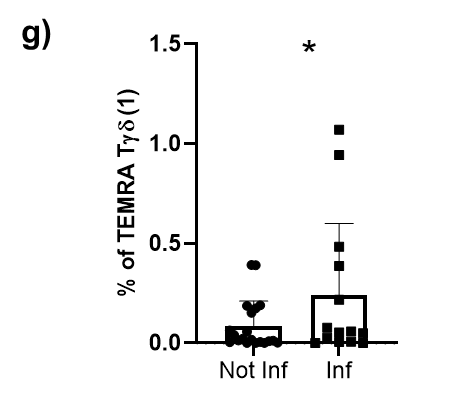


**Supplementary Fig. 5. Immunome differences before vaccination predicts SARS-CoV-2 infection.**

**(a)** Classical validation, as in Supplementary Fig. 1, of the significantly expressed populations in the volcano plot from Fig. 4b comparing the immune systems of all individuals before vaccination based on their subsequent infection following immunisation, and **(b)** their specific cell clusters. **(c)** Comparison of the significantly expressed populations in the volcano plot from Fig. 4d comparing the immune system of infected and non-infected healthy adults before vaccination, and **(d)** their specific cell clusters. **(e)** Analysis of the differentially expressed cell clusters in the volcano plot from Fig. 4e comparing infected and non-infected older adults before vaccination. **(f)** The proportion of the significantly expressed populations in the volcano plot from Fig. 4f comparing infected and non-infected oncohaematologic patients before vaccination, and **(g)** their specific clusters. In all cases, a t-test was performed where p < 0.05 was considered significant (*p < 0.05; **p < 0.01). A p-value between 0.05 and 0.1 was considered not significant (ns) but with a relevant trend (the exact p-value is shown). ‘Inf’ refers to subsequently infected individuals, while ‘Not Inf’ are those who were not infected.
